# Discordance Between BMI-Defined Overweight/Obesity and Waist-to-Height Ratio-Defined Central Obesity Among Adults with Type 2 Diabetes in Malawi: A Multicenter Cross-Sectional Study

**DOI:** 10.3390/diseases14090334

**Published:** 2026-09-13

**Authors:** Alexander Thomas Mboma, Elias Peter Mwakilama, Alexander Archippus Kalimbira, Duc Minh Cap, Tuyen Van Duong, Rong-Hong Hsieh

**Affiliations:** 1 School of Nutrition and Health Sciences, College of Nutrition, Taipei Medical University, Taipei 11031, Taiwan; da07111005@tmu.edu.tw (A.T.M.); da07113005@tmu.edu.tw (D.M.C.); 2 Department of Research and Development, Sub-Sahara Health and Research Institute, Lilongwe P.O. Box 40371, Malawi; emwakilama@unima.ac.mw; 3 Department of Mathematical Sciences, University of Malawi, Zomba P.O. Box 280, Malawi; 4 Department of Human Nutrition and Health Sciences, Bunda College, Lilongwe University of Agriculture & Natural Resources, Lilongwe P.O. Box 219, Malawi; akalimbira@luanar.ac.mw; 5 Faculty of Public Health, Haiphong University of Medicine and Pharmacy, Haiphong 180000, Vietnam

**Keywords:** type 2 diabetes mellitus, BMI-defined overweight/obesity, central obesity, waist circumference, waist-to-hip ratio, waist-to-height ratio, Malawi, late-evening eating behavior, physical activity, BMI-WHtR phenotypes

## Abstract

**Background:** Body mass index (BMI) does not capture abdominal fat distribution and may classify adiposity differently from central-obesity indicators. We quantified discordance between BMI-defined overweight/obesity and waist-to-height ratio (WHtR)-defined central obesity among adults with type 2 diabetes mellitus (T2DM) in Malawi. **Methods:** This multicenter cross-sectional study included 1356 adults attending diabetes clinics at three tertiary hospitals. Overweight/obesity was BMI ≥ 25 kg/m^2^. Central obesity was defined by waist circumference (WC ≥ 94/80 cm, men/women), waist-to-hip ratio (WHR ≥ 0.90/0.85), and WHtR ≥ 0.50. BMI and WHtR were cross-classified into four phenotypes, and agreement between the two classifications was summarized using observed agreement and Cohen’s kappa. Logistic and multinomial logistic regression models estimated (adjusted) odds ratios with 95% CIs; sensitivity analyses added study site as a fixed effect and modified Poisson models with robust variance provided adjusted prevalence ratios for the common binary outcomes. **Results:** Prevalence of BMI-defined overweight/obesity was 60.8%; central obesity was 67.5% (WC), 65.3% (WHR), and 80.1% (WHtR). Cross-classification showed 23.7% had central obesity only and 56.3% had combined overweight/obesity and central obesity; observed agreement between the two classifications was 71.8% and Cohen’s kappa was 0.35 (95% CI 0.30–0.40). Overweight/obesity was associated with older age (aOR 2.95), female sex (aOR 2.19), and physical inactivity (aOR 1.93). WC-defined central obesity was strongly associated with female sex (aOR 10.16), although the corresponding adjusted prevalence ratio was considerably smaller (aPR 2.25). WHR-defined central obesity was associated with older age, and anxiety. WHtR-defined central obesity was associated with older age, female sex, ever-married status, and late-evening eating behavior (aOR 1.36), although the association did not persist when study site was added. The combined phenotype was associated with age, sex, marital status, meal frequency, and physical inactivity. **Conclusions:** Overweight/obesity and central obesity were highly prevalent, with WHtR identifying the greatest burden and a substantial central-obesity-only phenotype that BMI alone would not detect. Incorporating WHtR alongside BMI may improve anthropometric assessment of adiposity in diabetes care, particularly among older and female patients.

## 1. Introduction

The global prevalence of type 2 diabetes mellitus (T2DM) has increased substantially, including across sub-Saharan Africa and Malawi [1], driven by rapid urbanization [2], dietary transition [3], reduced physical activity, and population aging [4]. Obesity is a key modifiable determinant of T2DM onset and progression and is strongly associated with hypertension, dyslipidemia, cardiovascular disease, and premature mortality [5,6,7,8]. Among people living with T2DM, excess adiposity may worsen glycemic control and amplify cardiometabolic risk through insulin resistance, chronic inflammation, and central and visceral fat accumulation [9,10,11]. Evidence from clinic-based settings further suggests that structural and behavioral barriers, particularly related to diet and physical activity may perpetuate excess adiposity, thereby undermining metabolic control despite pharmacological treatment [12].

Although body mass index (BMI) is widely used to classify overweight and obesity, it does not capture body fat distribution and may underestimate cardiometabolic risk among individuals with excess abdominal adiposity [13]. Consequently, individuals with BMI below the threshold for overweight may nevertheless have substantial abdominal adiposity. Complementary measures such as waist circumference (WC), waist-to-hip ratio (WHR), and waist-to-height ratio (WHtR) can improve risk stratification because abdominal adiposity is more strongly related to adverse metabolic outcomes than overall adiposity [14,15,16,17]. WHtR has been proposed as a practical screening metric that can identify elevated cardiometabolic risk even among individuals with BMI below conventional obesity thresholds [18]. Its simplicity and reliance on a commonly applied single cut-off across sexes makes WHtR particularly attractive for routine clinical settings in low-resource contexts.

Cross-classifying BMI and a central-adiposity measure identifies concordant and discordant phenotypes. Of particular clinical interest is the central-obesity-only phenotype, in which BMI is <25 kg/m^2^ but meets the criterion for central obesity. Such individuals would not be classified as having overweight/obesity by BMI alone. Conversely, some individuals may have BMI-defined overweight/obesity without central obesity. Behavioral and psychosocial factors may also differ across these phenotypes because meal timing, physical activity, sedentary behavior, depression, anxiety, and stress can be associated with adiposity and self-care among adults with T2DM [19,20,21,22].

Most studies in sub-Saharan Africa have reported BMI-defined overweight/obesity or central obesity separately, and evidence on the extent of discordance between the two classifications remains limited, particularly among adults receiving outpatient diabetes care in Malawi. The primary objective of this study was therefore to quantify classification concordance and discordance, by cross-classification and by a chance-corrected agreement statistic, between BMI-defined overweight/obesity and WHtR-defined central obesity among adults with T2DM attending tertiary outpatient diabetes clinics in Malawi. Secondary objectives were to estimate the prevalence of BMI-defined overweight/obesity and central obesity according to WC, WHR, and WHtR and to identify sociodemographic, clinical, behavioral, and psychological factors associated with the individual adiposity outcomes and BMI-WHtR phenotypes.

## 2. Materials and Methods

### 2.1. Study Design and Settings

This multicenter cross-sectional study was conducted between November 2024 and January 2025 at three purposively selected central hospitals representing Malawi’s three regions: Mzuzu Central Hospital (northern region), Kamuzu Central Hospital (central region), and Zomba Central Hospital (southern region). These facilities are tertiary-level government referral hospitals located in urban areas and serving surrounding district hospitals. All sites provide specialist outpatient care for chronic conditions, including routine diabetes clinics. Data were collected during routine outpatient diabetes clinic visits.

### 2.2. Study Population and Eligibility Criteria

Adults aged ≥18 years with a clinician-confirmed T2DM attending outpatient diabetes clinics were enrolled using consecutive sampling during the study period. Participants with prediabetes, type 1 diabetes or gestational diabetes were excluded. Additional exclusion criteria included a diabetes diagnosis duration of less than six months, critical illness, inpatient status, and cognitive impairment that precluded informed participation. All participants provided written informed consent.

### 2.3. Sample Size and Statistical Power

A formal a priori sample size was not calculated, as recruitment was based on consecutive clinic attendance during the study period. The achieved sample size (n = 1356) provided adequate precision for prevalence estimation, with a maximum 95% confidence interval half-width of approximately ±2.7% under conservative assumptions (*p* = 0.50) [23]. The large number of outcome events also supported stable multivariable and multinomial regression analyses of prespecified predictors. The study was not, however, powered for any individual subgroup, adiposity definition, or phenotype contrast; some phenotype categories are small, and estimates for those contrasts are correspondingly imprecise and are treated as exploratory.

### 2.4. Data Collection Procedures and Quality Assurance

Data were collected by trained nurse research assistants using a pretested, interviewer-administered questionnaire and standardized anthropometric measurement procedures. Data collectors received uniform training prior to study initiation, and measurement equipment was calibrated across study sites. All anthropometric measurements were taken once per participant following standardized positioning and measurement protocols to ensure consistency (implausible measurements were repeated, with the second value recorded). Because measurements were taken once, no formal intra-or inter-observer reliability coefficients were estimated; measurement consistency relied on uniform training, standardized protocols, and equipment calibration across sites.

#### 2.4.1. Sociodemographic and Clinical Characteristics

Sociodemographic variables included age group (18–39, 40–59, and 60–85 years), sex, marital status (never-married vs. ever-married), education level (pre-primary, primary, secondary, tertiary), and monthly household income reported in Malawi Kwacha (MWK) (≤MWK 150,000 vs. >MWK 150,000). Clinical variables included diabetes duration (<2 vs. ≥2 years), family history of diabetes (yes vs. no/don’t know), and diabetes management (lifestyle modification vs. medication).

#### 2.4.2. Behavioral and Psychological Characteristics

Total weekly meal frequency was calculated from reported breakfast, lunch and dinner consumption over the past 7 days. The variable is bounded above at 21 (three meals per day for seven days), and 866 participants (63.9%) reported exactly 21 meals, so three approximately equal groups could not be formed; the categories used (<20, 20, and >21 meals/week) are therefore defined by the structure of the variable rather than by a tertile split, and are not described as tertiles. Late-evening eating behavior was assessed as a binary variable (yes/no), defined as a composite of eating within two hours of bedtime and consuming snacks after dinner ≥ 3 times/week over the past 7 days [22]. This study-specific behavior measure derived from self-reported meal timing and post-dinner snacking items; it does not correspond to a validated diagnostic definition of night eating syndrome.

Physical activity was assessed using the International Physical Activity Questionnaire-Short Form (IPAQ-SF) and categorized according to World Health Organization (WHO) recommendations as meeting (≥600 MET-min/week) or not meeting recommended levels (<600 MET-min/week). Sedentary behavior was measured by IPAQ-SF sitting time items and categorized as <8 or ≥8 h per day, a commonly applied epidemiologic threshold in sedentary behavior [24].

Symptoms of depression, anxiety, and stress were screened using the Depression Anxiety Stress Scale-21 (DASS-21) [25]. The DASS-21 has demonstrated acceptable psychometric performance among postpartum women in Malawi [26], although it has not been specifically validated among Malawian adults with T2DM. Each item is rated on a 4-point Likert scale ranging from 0 (did not apply to me at all) to 3 (applied to me very much or most of the time). Each subscale (depression, anxiety, stress) comprises 7 items, yielding raw scores ranging from 0–21, which were multiplied by two to obtain comparable scores to the original 42-item scale (range 0–42) [27]. Standard cut-offs were applied: depression (DASS-D > 9), anxiety (DASS-A > 7), and stress (DASS-S > 14).

### 2.5. Anthropometric Measurements, Adiposity Outcomes and Phenotypes

Anthropometric measurements were performed using validated and recently calibrated equipment according to standardized operating procedures applied consistently across all the study sites. Standing height was measured using a standard hospital-grade stadiometer routinely used in Malawian hospitals. Participants were measured barefoot, standing upright with the head positioned in the Frankfort horizontal plane and height was recorded to the nearest 0.1 cm per WHO recommendations [28]. Body weight was measured using a calibrated digital scale, with participants barefoot and wearing light clothing and recorded to the nearest 0.1 kg [28]. Waist circumference was measured at the midpoint between the lower margin of the last palpable rib and the top of the iliac crest, and hip circumference at the widest point over the buttocks, using a non-stretchable measuring tape; both were recorded to the nearest 0.1 cm.

BMI was calculated as weight (kg) divided by height squared (m^2^); WHR as waist circumference (cm) divided by hip circumference (cm); and WHtR as waist circumference (cm) divided by height (cm).

BMI-defined overweight/obesity was defined as BMI ≥ 25 kg/m^2^. Central obesity by waist circumference was defined using the International Diabetes Federation (IDF) interim thresholds applied to Sub-Saharan African populations, ≥94 cm in men and ≥80 cm in women, and by the World Health Organization WHR thresholds of ≥0.90 in men and ≥0.85 in women [29]. Central obesity by WHtR was defined as WHtR ≥ 0.50, a commonly used cardiometabolic risk threshold [30]. No waist circumference, waist-to-hip ratio, or waist-to-height ratio cut-offs derived and validated in Malawian adults, or in comparable southern African adults with TD2M, are currently available, which is why international thresholds were applied; because the estimated prevalence of central obesity depends directly on the threshold selected, a sensitivity analysis across alternative WHtR threshold is reported (Supplementary Table S6). WHtR was selected for phenotype construction because it is height-standardized, applies a single commonly used threshold across sexes, and is feasible for routine clinical assessment.

BMI-WHtR phenotypes were constructed to define parsimonious, clinically interpretable adiposity subgroups aligned with cardiometabolic risk. Four mutually exclusive phenotypes were defined: (1) neither BMI-defined overweight/obesity nor central obesity (BMI < 25 kg/m^2^ and WHtR < 0.50); (2) BMI-defined overweight/obesity only (BMI ≥ 25 kg/m^2^ and WHtR < 0.50); (3) central obesity only (BMI < 25 kg/m^2^ and WHtR ≥ 0.50); and (4) combined BMI-defined overweight/obesity and central obesity (BMI ≥ 25 kg/m^2^ and WHtR ≥ 0.50). The central-obesity-only phenotype represented discordance in which WHtR identified central adiposity among participants not classified as overweight/obese by BMI.

### 2.6. Statistical Analysis

Participant characteristics were summarized as means (SD) for continuous variables and frequencies (%) for categorical variables. Prevalence estimates were calculated for BMI-defined overweight/obesity and central obesity by WC, WHR, WHtR. Group differences were assessed using Pearson’s chi-square test for categorical variables, with statistical significance set at *p* < 0.05. Agreement between BMI-defined overweight/obesity and WHtR-defined central obesity was quantified as the observed percentage agreement and Cohen’s kappa with a bias-corrected bootstrap 95% confidence interval (4000 replications). Kappa is reported as a descriptive, chance-corrected summary only; it does not establish that the two measures are interchangeable, because BMI and WHtR capture different dimensions of adiposity and their marginal prevalences differ substantially.

Factors associated with BMI-defined overweight/obesity and central obesity were identified using univariable and multivariable logistic regression models, with results presented as odds ratios (ORs) and adjusted odds ratios (aORs) with 95% confidence intervals (CIs). Multivariable models were built using a two-stage covariate strategy specified in advance. Age group, sex and marital status were entered into every model as core demographic confounders. Each remaining candidate covariate (education level, monthly income, diabetes duration, family history of diabetes, current treatment, meal frequency, late-evening eating behavior, physical activity, sedentary behavior, depression, anxiety, and stress) entered a given outcome’s adjusted model only where its univariable association with that outcome reached *p* < 0.20 on a likelihood-ratio test; for the multinomial phenotype outcome a stricter threshold of *p* < 0.10 was applied, because the BMI-only stratum contains only 61 participants and a larger adjustment set would be poorly identified against it. The screening *p*-values and the resulting adjustment set for every outcome are reported in Supplementary Table S3. Multicollinearity was assessed using generalized variance inflation factors (all ≤1.33) and model fit using the Hosmer–Lemeshow test and Nagelkerke R^2^ (Supplementary Table S13). Because BMI-defined overweight/obesity and central obesity were common outcomes (prevalence 60.8–80.1%), odds ratios overstate the magnitude of association relative to prevalence ratios; odds ratios are therefore interpreted strictly as measures of association, and adjusted prevalence ratios estimated by modified Poisson regression with robust (sandwich) variance, together with adjusted predicted prevalences obtained by marginal standardization, are reported alongside them (Supplementary Tables S4 and S5).

BMI-WHtR phenotypes were analyzed using univariable and multivariable multinomial logistic regression, with ‘neither BMI-defined overweight/obesity nor central obesity’ as the reference category. Results are presented as ORs and aORs with 95% CIs. There were no missing values on any analytic variable, so all models were fitted on the full analytic sample of 1356 participants with no cases dropped (Supplementary Table S12). Sensitivity analyses repeated every model with study site added as a fixed-effect covariate (Supplementary Table S2); examined alternative WHtR thresholds (Supplementary Table S6); examined alternative specifications of meal frequency, including a continuous form and two collapsed categorizations (Supplementary Table S9); applied Firth penalized estimation to the multinomial contrasts to address sparse-data bias (Supplementary Table S8); and compared central obesity among female participants aged < 50 and ≥ 50 years as a proxy for the menopausal transition (Supplementary Table S10). Study site was not included in the primary models because a fixed-effect specification with only three clusters is weakly identified; it is reported throughout as a sensitivity analysis. Analyses were conducted using complete-case data in IBM SPSS Statistics version 27.0 (IBM Corp., Armonk, NY, USA). The study is reported in accordance with the STROBE statement for cross-sectional studies, and the completed checklist is provided as Supplementary Table S14.

## 3. Results

### 3.1. Participant Characteristics and Prevalence of Adiposity Outcomes

Of 1489 patients screened for eligibility, 33 (2.2%) declined participation or did not provide consent, 1456 consented, and 100 were subsequently excluded on the prespecified criteria, leaving 1356 adults with clinician-confirmed T2DM in the analysis (Mzuzu n = 202; Kamuzu n = 599; Zomba n = 555). Participants were predominantly female (63.6%) with a mean (SD) age of 52.8 years (14.8) years; 42.4% were aged 40–59 years and 37.7% were aged 60–85 years. Most participants were ever-married (92.6%), had primary-level education (41.4%), and reported a monthly household income of ≤MWK 150,000 (68.7%) (Table 1).

The mean (SD) weight, height, BMI, WC, WHR, and WHtR were 68.49 (12.96) kg, 159.33 (8.80) cm, 27.13 (5.55) kg/m^2^, 93.39 (14.10) cm, 0.92 (0.17), and 0.59 (0.10), respectively (Table 1). BMI-defined overweight/obesity was present in 60.8% of participants. Central obesity prevalence was 67.5% by WC, 65.3% by WHR, and 80.1% by WHtR (Table 1). Most participants had diabetes duration ≥ 2 years (82.6%) and were managed with medication (97.6%). Overall, 86.0% met WHO physical activity recommendations and 8.6% reported sedentary time ≥ 8 h/day. Based on DASS-21 cut-offs, 27.1% screened positive for depression, 36.1% for anxiety, and 12.0% for stress.

### 3.2. BMI–WHtR Classification Concordance and Discordance

Cross-classification of BMI and WHtR revealed marked discordance in adiposity classification. Overall, 209 participants (15.4%) met neither the BMI-defined overweight/obesity nor the central obesity criterion, 61 (4.5%) met the BMI-defined overweight/obesity criterion only, 322 (23.7%) met the central obesity criterion only, and 764 (56.3%) met both. Observed agreement between the two classifications was 71.8% and Cohen’s kappa was 0.35 (95% CI 0.30–0.40), indicating only modest chance-corrected agreement. Nearly one-quarter of participants therefore had WHtR-defined central obesity despite a BMI below the overweight threshold, whereas BMI-defined overweight/obesity without WHtR-defined central obesity was uncommon. The discordance was present at all three hospitals and was robust to the WHtR threshold applied. At the most conservative threshold examined (≥0.55), 14.2% of participants still had the central-obesity-only phenotype and 11.1% the BMI-only phenotype (Supplementary Tables S1 and S6).

### 3.3. Factors Associated with BMI-Defined Overweight/Obesity and Central Obesity

#### 3.3.1. BMI-Defined Overweight/Obesity

After multivariable adjustment, BMI-defined overweight/obesity was associated with older age, female sex, ever-married status, higher weekly meal frequency, and not meeting the WHO physical activity recommendations. Compared with participants aged 18–39 years, odds were higher among those aged 40–59 years (aOR 2.30, 95% CI 1.66–3.18) and 60–85 years (aOR 2.95, 95% CI 2.11–4.13). Women had higher odds than men (aOR 2.19, 95% CI 1.72–2.79), and ever-married participants had higher odds than never-married participants (aOR 1.65, 95% CI 1.03–2.64). Compared with participants reporting fewer than 20 meals per week, odds were higher among those reporting 20 meals/week (aOR 2.72, 95% CI 1.29–5.73) and 21 meals/week (aOR 1.47, 95% CI 1.15–1.89). Participants not meeting WHO physical activity recommendations had higher odds of overweight/obesity than those who met them (aOR 1.93, 95% CI 1.34–2.78) (Table 2).

#### 3.3.2. Central Obesity

WC-defined central obesity was associated after multivariable adjustment with older age, female sex, and ever-married status. Odds were higher among participants aged 40–59 years (aOR 2.76, 95% CI 1.90–4.01) and 60–85 years (aOR 2.67, 95% CI 1.82–3.93) relative to those aged 18–39 years. Women had markedly higher odds than men (aOR 10.16, 95% CI 7.67–13.45), and ever-married participants had higher odds than never-married participants (aOR 1.96, 95% CI 1.16–3.32) (Table 2). The very large odds ratio for female sex should not be read as a prevalence contrast: the corresponding adjusted prevalence ratio was 2.25 (95% CI 2.01–2.53), and the adjusted predicted prevalence of WC-defined central obesity was 84.3% in women compared with 37.7% in men (Supplementary Tables S4 and S5).

WHR-defined central obesity was associated after multivariable adjustment with older age, female sex, and anxiety symptoms. Odds were higher among participants aged 40–59 years (aOR 1.66, 95% CI 1.20–2.30) and 60–85 years (aOR 2.11, 95% CI 1.51–2.94). Women had higher odds than men (aOR 1.92, 95% CI 1.51–2.44), and participants screening positive for anxiety had higher odds than those screening negative (aOR 1.44, 95% CI 1.04–1.99) (Table 2).

WHtR-defined central obesity was associated after multivariable adjustment with older age, female sex, ever-married status, and late-evening eating behavior. Odds were higher among participants aged 40–59 years (aOR 2.85, 95% CI 1.98–4.09) and 60–85 years (aOR 3.07, 95% CI 2.11–4.47). Women had higher odds than men (aOR 2.77, 95% CI 2.08–3.68), ever-married participants had higher odds than never-married participants (aOR 2.12, 95% CI 1.31–3.43), and participants reporting late-evening eating had higher odds than those who did not (aOR 1.36, 95% CI 1.02–1.80) (Table 2).

### 3.4. Factors Associated with BMI-WHtR Phenotypes

Factors associated with each BMI-WHtR phenotype, relative to participants meeting neither criterion, are shown in Table 3.

In adjusted multinomial models, older age and female sex remained associated with higher odds of both central obesity only and combined phenotypes relative to neither condition. Compared with participants aged 18–39 years, odds of the central-obesity-only phenotype were higher among those aged 40–59 years (aOR 2.24, 95% CI 1.42–3.54) and 60–85 years (aOR 2.03, 95% CI 1.25–3.31); odds of the combined phenotype were higher in both age groups as well (40–59 years: aOR 4.09, 95% CI 2.65–6.33; 60–85 years: aOR 4.94, 95% CI 3.14–7.78). Women had higher odds than men of the central-obesity-only phenotype (aOR 2.40, 95% CI 1.66–3.47) and the combined phenotype (aOR 4.04, 95% CI 2.87–5.68). Ever-married participants had higher odds than never-married participants of the central-obesity-only phenotype (aOR 2.63, 95% CI 1.41–4.89) and the combined phenotype (aOR 2.59, 95% CI 1.48–4.55). Reporting 20 meals/week was associated with higher odds of the BMI-defined overweight/obesity-only phenotype (aOR 7.62, 95% CI 1.47–39.66) and the combined phenotype (aOR 3.88, 95% CI 1.04–14.49) relative to fewer than 20 meals/week; only 46 participants fall in this category, however, and these estimates are very imprecise. Not meeting WHO physical activity recommendations was associated after multivariable adjustment with higher odds of the combined phenotype (aOR 1.97, 95% CI 1.13–3.45) (Table 3).

### 3.5. Sensitivity Analyses

The study site was associated with adiposity classification. The phenotype distribution differed across the three hospitals (χ^2^ = 67.84, df = 6, *p* < 0.001), and BMI-defined overweight/obesity ranged from 54.4% at Kamuzu to 66.8% at Mzuzu while WHtR-defined central obesity ranged from 71.6% at Kamuzu to 88.3% at Zomba (Supplementary Table S1). Repeating every model in Table 2 and Table 3 with study site added as a fixed-effect covariate left the demographic and behavioral associations materially unchanged. Two associations did not survive site adjustment: late-evening eating behavior and WHtR-defined central obesity was attenuated to the null (aOR 1.12, 95% CI 0.83–1.51, compared with 1.36, 95% CI 1.02–1.80 in the primary model), and the 20 meals/week contrast for the combined phenotype crossed the null (aOR 3.78, 95% CI 0.96–14.80, compared with 3.88, 95% CI 1.04–14.49). Study site was itself associated with WHtR-defined central obesity (Zomba vs. Mzuzu aOR 1.90, 95% CI 1.17–3.07) and with the central-obesity-only and combined phenotypes (Supplementary Table S2).

Because BMI-defined overweight/obesity and central obesity are common outcomes, adjusted prevalence ratios were also estimated by modified Poisson regression with robust variance. These were substantially closer to the null than the corresponding odds ratios and preserved the direction of every association. Statistical significance was preserved in all but one instance: ever-married status and BMI-defined overweight/obesity was significant on the odds scale (aOR 1.65, *p* = 0.035) but not on the prevalence scale (aPR 1.26, 95% CI 0.99–1.61, *p* = 0.062) (Supplementary Table S4), and adjusted predicted prevalences are reported alongside these results (Supplementary Table S5). The cross-classification finding was robust to the WHtR threshold applied (Supplementary Table S6). Model diagnostics are reported in Supplementary Table S13: the three binary models for BMI-defined overweight/obesity, WC-defined and WHR-defined central obesity showed no evidence of lack of fit, whereas the WHtR model did (Hosmer–Lemeshow χ^2^ = 18.70. df = 8, *p* = 0.017), so the covariate associations for that outcome are interpreted with corresponding caution. Estimates for the BMI-only phenotype remained imprecise under penalized estimation of the corresponding binary contrasts; for the 20 meals/week contrasts the penalized estimate was larger, not smaller, than the maximum-likelihood estimate (8.79, 95% CI 1.61–48.03, against 7.62, 95% CI 1.47–39.66), the penalized estimate for the same contrast against the combined phenotype no longer reached significance (*p* = 0.055 against *p* = 0.043) (Supplementary Table S8), and the meal-frequency association with BMI-defined overweight/obesity persisted when the small 20 meals/week category was collapsed in either direction but not when meal frequency was modeled continuously (Supplementary Table S9). Among female participants, central obesity was more prevalent in those aged ≥ 50 years than in younger women for all three indicators (Supplementary Table S10).

## 4. Discussion

This multicenter study quantified concordance and discordance between BMI-defined overweight/obesity and central obesity, and identified sociodemographic, clinical, behavioral, and psychological correlates of these adiposity outcomes among adults with T2DM attending tertiary outpatient diabetes clinics in Malawi. The principal finding was substantial discordance between BMI-defined overweight/obesity and central obesity: while 60.8% of participants had BMI-defined overweight/obesity, central obesity was present in 67.5% to 80.1% of participants depending on the indicator used, with waist-to-height ratio (WHtR) identifying the greatest burden. Cross-classification of BMI and WHtR showed that nearly one-quarter of participants (23.7%) had central obesity despite a BMI < 25 kg/m^2^, whereas only 4.5% had BMI-defined overweight/obesity without central obesity. Observed agreement between the two classifications was 71.8%, but chance-corrected agreement was modest (kappa 0.35, 95% CI 0.30–0.40), and the discordance persisted across all three hospitals and across a range of alternative WHtR thresholds. These findings indicate that BMI and WHtR capture partly distinct adiposity phenotypes, and that reliance on BMI alone would substantially underestimate the burden of central adiposity in this population.

The prevalence of overweight/obesity observed in this study is comparable to reports from other T2DM populations in low and middle income settings, including Thailand (51.1%) [31], India (58.7% BMI-defined overweight/obesity and 81.8% abdominal obesity) [32], and East African settings such as Uganda (28% overweight) [33] and Tanzania (44.8% overweight and 33.9% obesity) [33]. Regional evidence also suggests a similarly high burden among adults with diabetes across Africa; a recent review estimated pooled prevalences of 35.6% for overweight and 25.6% for obesity, with a combined prevalence of 61.4%, and marked geographic variation (56.9% in East Africa to 88.5% in southern Africa) [34]. Consistent with our findings, central obesity has also been reported to be common among adults with diabetes in Africa, for example 55.4% in Nigeria [35].

These findings reinforce well-recognized limitations of BMI as a sole indicator of obesity. BMI does not distinguish between fat and lean mass or capture regional fat distribution, which is particularly relevant in populations where visceral and abdominal fat accumulation drive cardiometabolic risk. This concern is clinically relevant because “normal-weight central obesity” has been associated with adverse cardiovascular risk profiles, including hypertension and dyslipidemia when compared with normal weight without central adiposity [36]. Recent conceptual advances in obesity diagnosis emphasize the need for a more nuanced diagnostic framework that emphasizes a body composition-oriented approach supported by multiple complementary measurement modalities, rather than reliance on BMI alone [37]. Similarly, population-based body composition data from the National Health and Nutrition Examination Survey (NHANES) illustrate how poorly weight-for-height alone characterizes fat distribution, supporting the use of complementary indicators such as waist circumference, waist-to-hip ratio, and WHtR [38].

A key contribution of this study is the characterization of BMI-WHtR phenotypes in a routine diabetes care population in Malawi. Nearly one in four participants (23.7%) exhibited central obesity despite having a BMI < 25 kg/m^2^, representing a clinically important “central-obesity-only” phenotype that would likely be missed if assessment were limited to BMI. In contrast, only a small proportion had BMI-defined overweight/obesity without central obesity, whereas more than half had the combined phenotype. These distributions highlight that central adiposity is the dominant obesity pattern among adults with T2DM in this setting and support the incorporation of waist-to-height ratio into routine clinical assessment as a simple, low-cost screening tool requiring only a tape measure and height. Similar patterns of BMI-independent central obesity have been reported in other populations with diabetes, reinforcing the broader relevance of these findings [39,40].

Older age and female sex emerged as the most consistent and robust correlates of both BMI-defined overweight/obesity and central obesity across all anthropometric indicators examined; BMI, WC, WHR, and WHtR. This pattern is consistent with evidence from sub-Saharan Africa and other regions, where studies among adults with T2DM and general populations have repeatedly shown a higher burden of obesity among older individuals and women [33,34,41,42]. The observed age gradient may reflect cumulative lifetime exposure to obesogenic environments [43,44], together with age-related physiological changes such as declines in physical activity [43], loss of skeletal muscle mass, and reductions in basal energy expenditure, which collectively favor fat accumulation and redistribution toward the abdominal region [45,46].

Female participants had substantially higher odds of both BMI-defined overweight/obesity and central obesity, including a particularly strong association with WC-defined central obesity. Similar sex differentials have been reported in African populations, where women consistently exhibit higher overall and central adiposity than men, particularly in mid-to-late adulthood [47,48,49]. The markedly higher odds of central obesity among women, observed across WC, WHR, and WHtR-based definitions, suggest a true excess burden of abdominal adiposity rather than a methodological artifact of sex-specific cut-offs [50]. Evidence from African and non-African settings further indicates that women may experience disproportionate cardiometabolic consequences of central fat accumulation, underscoring the clinical relevance of these observations [51]. Collectively, these findings underscore the importance of prioritizing older adults and women for targeted obesity prevention and management strategies within diabetes care.

Ever-married status was also associated with BMI-defined overweight/obesity and with central obesity defined by WC and WHtR. This pattern may reflect differences in household food availability, social eating practices, and time constraints related to family responsibilities, which can influence dietary habits and physical activity. While marital status may also be a marker of age and broader socioeconomic patterns, the persistence of associations in adjusted models suggests that household and social-context factors warrant consideration in designing clinic-based counseling and community-linked interventions.

Several behavioral factors were associated with the adiposity outcomes examined. Higher weekly meal frequency was associated with BMI-defined overweight/obesity and with combined phenotype, a pattern that would be consistent with higher total energy intake, although the direction of the association cannot be established from these data. One cross-sectional study reported the opposite pattern, with higher eating frequency associated with lower adiposity [52]. Interpretation of the meal frequency findings warrants caution. Weekly meal frequency is bounded above at 21, and only 46 participants reported exactly 20 meals per week, so estimates for that category are unstable. In sensitivity analyses, the association with BMI-defined overweight/obesity was preserved when this small category was collapsed in either direction, but was not statistically significant when meal frequency was modeled continuously (Supplementary Table S9), so the finding is best regarded as suggestive rather than established. Nonetheless, the overall pattern aligns with evidence that eating frequency, in the absence of portion control and diet quality considerations, may promote positive energy balance and weight gain [53].

Not meeting WHO physical activity recommendations was associated after multivariable adjustment with BMI-defined overweight/obesity and with greater odds of combined phenotype, reinforcing the central role of physical activity promotion within diabetes management. This finding is consistent with prior studies from sub-Saharan Africa and other low- and middle-income settings, which demonstrate that insufficient physical activity is strongly linked to both general and central obesity among adults with T2DM [54,55]. Although most participants reported meeting WHO physical activity recommendations, over-reporting due to recall or social desirability bias cannot be excluded. Even so, the observed associations suggest that relatively modest differences in activity levels may have meaningful implications for adiposity patterns in this clinical population [56,57,58,59].

Late-evening eating behavior was associated with central obesity assessed by WHtR (aOR 1.36, 95% CI 1.02–1.80), supporting the plausibility that meal timing behaviors are linked to abdominal adiposity. Experimental and observational studies suggest that late eating may promote central fat accumulation through mechanisms involving circadian misalignment [60], altered substrate utilization, impaired glucose metabolism, and dysregulation of appetite-related hormones [61,62]. This association should, however, be interpreted with caution. It was the only finding in the study that did not persist when study site was added as a covariate (aOR 1.12, 95% CI 0.83–1.51; Supplementary Table S2), and the reason is visible in the data: Zomba Central Hospital had both the highest proportion of participants reporting late-evening eating (65.0%, compared with 38.1% at Mzuzu and 43.7% at Kamuzu) and the highest prevalence of WHtR-defined central obesity (88.3%), so the association is at least partly confounded by recruitment site. The measurement used here is also a study-specific composite of self-reported meal timing and post-dinner snacking rather than a validated definition of night eating syndrome. Notably, the association was observed for WHtR rather than BMI, consistent with the broader pattern in this study that behavioral factors relate more closely to abdominal adiposity which BMI-based screening may not adequately capture [63]. Meal timing and evening snacking are plausible topics for nutrition counselling in diabetes clinics, but this particular association requires confirmation in data that can separate behavior from setting.

Among psychosocial correlates of central obesity, anxiety was associated with central obesity defined by WHR in adjusted analysis (aOR 1.44, 95% CI 1.04–1.99), the only psychological measure to enter any of the adjusted models. This relationship may reflect bidirectional pathways linking psychological distress and obesity, including stress-related eating behaviors, sleep disturbance, and reduced engagement in physical activity [64,65]. However, the lack of consistent associations across all central obesity indicators and the cross-sectional nature of the data suggest that this finding should be interpreted cautiously. Nonetheless, given the high prevalence of anxiety symptoms observed in this sample and evidence that mental health influences adherence to lifestyle and self-care behaviors, integrating routine screening for common mental health conditions within diabetes care may support both psychological wellbeing and effective obesity management.

Adiposity classification also varied by recruitment site. The phenotype distribution differed significantly across the three hospitals (χ^2^ = 67.84, df = 6, *p* < 0.001), and in sensitivity models that added study site as a fixed effect, participants at Zomba Central Hospital had higher odds of WHtR-defined central obesity than those at Mzuzu Central Hospital (aOR 1.90, 95% CI 1.17–3.07), while those at Kamuzu Central Hospital had lower odds (aOR 0.60, 95% CI 0.39–0.92). The demographic and behavioral associations reported in Table 2 and Table 3 were materially unchanged by site adjustment, with the single exception of late-evening eating discussed above, and discordance between BMI and WHtR was present at every site, so the central finding is not generated by any one hospital. The site differences themselves are more plausibly explained by differences in referral catchment and local population characteristics than by measurement, but they indicate that a single national estimate of adiposity burden among adults with T2DM in Malawi would conceal meaningful regional variation.

Because women accounted for 63.6% of the sample and 63.3% of those women were aged ≥ 50 years, we examined central obesity by age within the female subgroup. Prevalence was higher in women aged ≥ 50 years than in younger women for every indicator (WC 87.5% vs. 80.1%; WHR 74.4% vs. 66.1%; WHtR 89.6% vs. 81.6%), and the differences persisted after adjustment for marital status and site (WHtR aOR 1.72, 95% CI 1.14–2.60) (Supplementary Table S10). This pattern is compatible with the redistribution of body fat toward the abdomen that accompanies the menopausal transition [66], although menopausal status was not ascertained and age ≥ 50 years is only a proxy for it.

### Strengths and Limitations

This study has several strengths, firstly, it included a large sample drawn from three of the four tertiary referral hospitals in Malawi, enhancing statistical power and providing multicenter evidence from routine diabetes care settings. Secondly, selection bias was minimized through consecutive recruitment of eligible clinic attendees. Thirdly, measurement quality was strengthened by the use of standardized operating procedures, calibrated equipment, and uniform training of data collectors across all study sites, reducing inter-site variability and measurement error. Collectively, these strengths support the internal validity of the study and the robustness of the anthropometric assessments.

Despite these strengths, this study also has the following limitations: First, the cross-sectional design precludes causal inference regarding behavioral and psychosocial correlates of obesity. Second, facility-based recruitment from tertiary referral clinics may limit generalizability to all adults with T2DM in Malawi, including those managed in primary care settings or not engaged in care. Third, key behavioral measures (meal frequency, late-evening eating behavior, physical activity, and sedentary time) were self-reported and may be affected by recall and social desirability bias, and anthropometric measurements were taken once and no intra- or inter-observer reliability coefficients were estimated, so misclassification of participants lying close to a threshold cannot be excluded. Fourth, no reference measure of body composition, such as dual-energy X-ray absorptiometry, magnetic resonance imaging, or bioelectrical impedance analysis, was available, so this study compares pragmatic anthropometric classifications with one another and cannot establish which of them more accurately reflects visceral or total adiposity. Fifth, the thresholds applied were derived in other populations, and the estimated prevalence of central obesity depends directly on the cut-off selected, as the threshold sensitivity analysis demonstrates. Sixth, the meal frequency categorization included a very small middle category (n = 46), and estimates involving this group, particularly in the phenotype models, should be interpreted cautiously. Seventh, no adjustment was made for multiple comparisons across the four adiposity outcomes, and the model for WHtR-defined central obesity showed evidence of lack of fit on the Hosmer–Lemeshow test (*p* = 0.017; Supplementary Table S13); individual secondary associations should therefore be read as exploratory. Finally, study site was handled as a parallel fixed-effect analysis rather than entered into the primary models, because the primary models were specified to describe the pooled tertiary-clinic population; the site-adjusted estimates are nonetheless reported for every model, and the association, which may introduce measurement error. Fourth, the meal frequency categorization included a very small middle category, and estimates involving this group (particularly in phenotype models) should be interpreted cautiously. Finally, although the study was multicenter, analyses did not explicitly account for clustering by site, which may have influenced precision; future studies should consider site-adjusted models or robust approaches as feasible.

## 5. Conclusions

Central obesity was substantially more prevalent than BMI-defined overweight/obesity among adults with T2DM attending tertiary outpatient care in Malawi, with waist-to-height ratio identifying the greatest burden. Nearly one-quarter of participants had a central-obesity-only phenotype that would be missed using BMI alone, indicating that BMI and WHtR provide complementary rather than interchangeable information. Older age, female sex, and modifiable behavioral factors, including physical activity, higher meal frequency, and late-evening eating behavior, were associated with greater odds of adiposity phenotypes. Incorporating routine assessment of central adiposity, particularly using WHtR, alongside targeted lifestyle and psychosocial support, may improve anthropometric assessment of adiposity in diabetes care in Malawi. Whether it also improves cardiometabolic risk stratification would require validation against independent clinical or metabolic outcomes, which this study did not measure.

## Figures and Tables

**Table 1:** Participant characteristics and prevalence of BMI-defined overweight/obesity and central obesity (n = 1356).

Variables	Total, n (%) or Mean (SD)	BMI-Defined Overweight/Obesity, n (%)	Central Obesity (WC), n (%)	Central Obesity (WHR), n (%)	Central Obesity (WHtR), n (%)
**Overall**	1356 (100)	825 (60.8)	915 (67.5)	885 (65.3)	1086 (80.1)
**Socio-demographics**					
Age group (years)					
18–39	270 (19.9)	102 (37.8)	124 (45.9)	137 (50.7)	160 (59.3)
40–59	575 (42.4)	365 (63.5)	422 (73.4)	383 (66.6)	488 (84.9)
60–85	511 (37.7)	358 (70.1)	369 (72.2)	365 (71.4)	438 (85.7)
Sex					
Male	494 (36.4)	238 (48.2)	184 (37.2)	270 (54.7)	339 (68.6)
Female	862 (63.6)	587 (68.1)	731 (84.8)	615 (71.3)	747 (86.7)
Marital status					
Never-married	100 (7.4)	39 (39.0)	48 (48.0)	54 (54.0)	56 (56.0)
Ever-married	1256 (92.6)	786 (62.6)	867 (69.0)	831 (66.2)	1030 (82.0)
Education level					
Pre-primary	123 (9.1)	75 (61.0)	96 (78.0)	92 (74.8)	101 (82.1)
Primary	561 (41.4)	343 (61.1)	385 (68.6)	377 (67.2)	452 (80.6)
Secondary	528 (38.9)	314 (59.5)	338 (64.0)	325 (61.6)	413 (78.2)
Tertiary	144 (10.6)	93 (64.6)	96 (66.7)	91 (63.2)	120 (83.3)
Monthly household income (MWK)					
≤150,000	931 (68.7)	566 (60.8)	625 (67.1)	617 (66.3)	740 (79.5)
>150,000	425 (31.3)	259 (60.9)	290 (68.2)	268 (63.1)	346 (81.4)
**Anthropometric indicators, Mean (SD)**					
Weight (kg)	68.49 (12.96)	74.63 (11.75)	71.68 (12.79)	69.59 (13.04)	70.19 (13.03)
Height (cm)	159.33 (8.80)	156.62 (8.38)	158.30 (8.60)	158.63 (8.86)	158.03 (8.63)
BMI (kg/m^2^)	27.13 (5.55)	30.44 (4.42)	28.74 (5.42)	27.81 (5.65)	28.22 (5.42)
WC (cm)	93.39 (14.10)	98.43 (13.00)	100.21 (11.11)	98.47 (12.76)	97.99 (11.63)
WHR	0.92 (0.17)	0.93 (0.16)	0.95 (0.18)	0.99 (0.17)	0.95 (0.17)
WHtR	0.59 (0.10)	0.63 (0.09)	0.64 (0.08)	0.62 (0.09)	0.62 (0.08)
**Clinical characteristics, n (%)**					
Diabetes duration (years)					
<2	236 (17.4)	137 (58.1)	158 (66.9)	158 (66.9)	182 (77.1)
≥2	1120 (82.6)	688 (61.4)	757 (67.6)	727 (64.9)	904 (80.7)
Family history of diabetes					
No/don’t know	768 (56.6)	455 (59.2)	500 (65.1)	491 (63.9)	613 (79.8)
Yes	588 (43.4)	370 (62.9)	415 (70.6)	394 (67.0)	473 (80.4)
Diabetes management					
Lifestyle modification	32 (2.4)	20 (62.5)	23 (71.9)	17 (53.1)	26 (81.2)
Medication	1324 (97.6)	805 (60.8)	892 (67.4)	868 (65.6)	1060 (80.1)
**Behavioral factors, n (%)**					
Total meal frequency per week					
<20 meals/week	444 (32.7)	240 (54.1)	295 (66.4)	291 (65.5)	355 (80.0)
20 meals/week	46 (3.4)	35 (76.1)	35 (76.1)	27 (58.7)	39 (84.8)
21 meals/week	866 (63.9)	550 (63.5)	585 (67.6)	567 (65.5)	692 (79.9)
Late-evening eating behavior					
No	656 (48.4)	396 (60.4)	436 (66.5)	417 (63.6)	509 (77.6)
Yes	700 (51.6)	429 (61.3)	479 (68.4)	468 (66.9)	577 (82.4)
Physical activity (Meets WHO recommendations)					
Yes (≥600 MET-min/week)	1166 (86.0)	682 (58.5)	774 (66.4)	757 (64.9)	923 (79.2)
No (<600 MET-min/week)	190 (14.0)	143 (75.3)	141 (74.2)	128 (67.4)	163 (85.8)
Sedentary behavior (hrs/day)					
<8	1240 (91.4)	755 (60.9)	837 (67.5)	812 (65.5)	996 (80.3)
≥8	116 (8.6)	70 (60.3)	78 (67.2)	73 (62.9)	90 (77.6)
**Psychological distress (DASS-21), n (%)**					
Depression					
No (DASS-D ≤ 9)	988 (72.9)	609 (61.6)	676 (68.4)	634 (64.2)	793 (80.3)
Yes (DASS-D > 9)	368 (27.1)	216 (58.7)	239 (64.9)	251 (68.2)	293 (79.6)
Anxiety					
No (DASS-A ≤ 7)	866 (63.9)	528 (61.0)	585 (67.6)	543 (62.7)	691 (79.8)
Yes (DASS-A > 7)	490 (36.1)	297 (60.6)	330 (67.3)	342 (69.8)	395 (80.6)
Stress					
No (DASS-S ≤ 14)	1193 (88.0)	723 (60.6)	811 (68.0)	775 (65.0)	958 (80.3)
Yes (DASS-S > 14)	163 (12.0)	102 (62.6)	104 (63.8)	110 (67.5)	128 (78.5)

The total column reports n (%) of the full sample (n = 1356); values in the adiposity outcome columns report n/N (%) within each subgroup. BMI-defined overweight/obesity: BMI ≥ 25 kg/m^2^. Central obesity: WC ≥ 94 cm in men and ≥80 cm in women; WHR ≥ 0.90 in men and ≥0.85 in women; WHtR ≥ 0.50. Denominators, prevalences, and omnibus chi-square *p*-values for every characteristic across all four adiposity definitions are reported in Supplementary Table S11. During the study period, the average exchange rate was approximately MWK 1751 = USD 1. Income categories are presented in Malawian Kwacha as reported by participants. Abbreviations: BMI, body mass index; WC, waist circumference; WHR, waist-to-hip ratio; WHtR, waist-to-height ratio; MET, metabolic equivalent of task; DASS-21, Depression Anxiety Stress Scales-21; WHO, World Health Organization.

**Table 2:** Factors associated with BMI-defined overweight/obesity and central obesity: results of univariable and multivariable logistic regression analyses (n = 1356).

Variable	BMI-Defined Overweight/Obesity		Central Obesity (WC)		Central Obesity (WHR)		Central Obesity (WHtR)	
	OR (95% CI)	aOR (95% CI)	OR (95% CI)	aOR (95% CI)	OR (95% CI)	aOR (95% CI)	OR (95% CI)	aOR (95% CI)
**Socio-demographics**								
Age group (years)								
18–39	Ref	Ref	Ref	Ref	Ref	Ref	Ref	Ref
40–59	2.86 (2.12–3.86) ***	2.30 (1.66–3.18) ***	3.25 (2.40–4.40) ***	2.76 (1.90–4.01) ***	1.94 (1.44–2.60) ***	1.66 (1.20–2.30) **	3.86 (2.76–5.38) ***	2.85 (1.98–4.09) ***
60–85	3.85 (2.82–5.25) ***	2.95 (2.11–4.13) ***	3.06 (2.25–4.16) ***	2.67 (1.82–3.93) ***	2.43 (1.79–3.30) ***	2.11 (1.51–2.94) ***	4.13 (2.92–5.84) ***	3.07 (2.11–4.47) ***
Sex								
Male	Ref	Ref	Ref	Ref	Ref	Ref	Ref	Ref
Female	2.30 (1.82–2.88) ***	2.19 (1.72–2.79) ***	9.40 (7.25–12.20) ***	10.16 (7.67–13.45) ***	2.07 (1.64–2.60) ***	1.92 (1.51–2.44) ***	2.97 (2.26–3.90) ***	2.77 (2.08–3.68) ***
Marital status								
Never-married	Ref	Ref	Ref	Ref	Ref	Ref	Ref	Ref
Ever-married	2.61 (1.72–3.97) ***	1.65 (1.03–2.64) *	2.42 (1.60–3.64) ***	1.96 (1.16–3.32) *	1.67 (1.11–2.51) **	1.24 (0.78–1.95)	3.58 (2.35–5.45) ***	2.12 (1.31–3.43) **
Education level		-						-
Pre-primary	Ref		Ref	Ref	Ref	Ref	Ref	
Primary	1.01 (0.67–1.50)		0.62 (0.39–0.98) *	0.72 (0.42–1.22)	0.69 (0.44–1.08)	0.74 (0.47–1.16)	0.90 (0.54–1.50)	
Secondary	0.94 (0.62–1.40)		0.50 (0.32–0.80) **	0.99 (0.58–1.71)	0.54 (0.35–0.84) **	0.71 (0.45–1.12)	0.78 (0.47–1.30)	
Tertiary	1.17 (0.70–1.92)		0.53 (0.33–0.98) *	1.37 (0.72–2.58)	0.58 (0.34–0.98) *	0.82 (0.47–1.42)	1.09 (0.58–2.06)	
Monthly household income (MWK)		-		-		-		-
≤150,000	Ref		Ref		Ref		Ref	
>150,000	1.01 (0.80–1.27)		1.05 (0.82–1.35)		0.87 (0.68–1.10)		1.13 (0.85–1.51)	
**Clinical characteristics**								
Duration of diabetes (years)		-		-		-		-
<2	Ref		Ref		Ref		Ref	
≥2	1.15 (0.87–1.53)		1.03 (0.76–1.39)		0.91 (0.68–1.23)		1.24 (0.89–1.74)	
Family history of diabetes						-		-
No/don’t know	Ref	Ref	Ref	Ref	Ref		Ref	
Yes	1.17 (0.93–1.46)	1.02 (0.81–1.29)	1.29 (1.02–1.62) *	0.94 (0.71–1.24)	1.15 (0.91–1.44)		1.04 (0.79–1.36)	
Current treatment		-		-				-
Lifestyle modification	Ref		Ref		Ref	Ref	Ref	
Medication	0.93 (0.45–1.92)		0.81 (0.37–1.76)		1.68 (0.83–3.39)	1.55 (0.74–3.23)	0.93 (0.38–2.27)	
**Behavioral factors**								
Total meal frequency per week				-		-		-
<20 meals/week	Ref	Ref	Ref		Ref		Ref	
20 meals/week	2.71 (1.34–5.46) **	2.72 (1.29–5.73) **	1.61 (0.79–3.25)		0.75 (0.40–1.39)		1.40 (0.61–3.23)	
21 meals/week	1.48 (1.17–1.87) ***	1.47 (1.15–1.89) **	1.05 (0.83–1.34)		1.00 (0.78–1.27)		1.00 (0.75–1.33)	
Late-evening eating behavior		-		-		-		
No	Ref		Ref		Ref		Ref	Ref
Yes	1.04 (0.84–1.29)		1.09 (0.87–1.37)		1.16 (0.92–1.45)		1.36 (1.07–1.77) *	1.36 (1.02–1.80) *
Physical Activity (Meets WHO recommendations)								
Yes (≥600 MET-min/week)	Ref	Ref	Ref	Ref	Ref	-	Ref	Ref
No (<600 MET-min/week)	2.16 (1.52–3.06) ***	1.93 (1.34–2.78) ***	1.46 (1.03–2.06) *	1.33 (0.89–1.99)	1.12 (0.81–1.55)		1.59 (1.03–2.45) *	1.43 (0.90–2.25)
Sedentary behavior (hrs./day)								
<8	Ref	-	Ref	-	Ref	-	Ref	-
≥8	0.98 (0.66–1.44)		0.99 (0.66–1.48)		0.90 (0.60–1.33)		0.85 (0.54–1.34)	
Psychological distress symptoms (DASS-21)								
Depression		-		-				-
No (DASS-D ≤ 9)	Ref		Ref		Ref	Ref	Ref	
Yes (DASS-D > 9)	0.88 (0.69–1.13)		0.86 (0.66–1.10)		1.20 (0.93–1.55)	0.94 (0.66–1.33)	0.96 (0.71–1.29)	
Anxiety		-		-				-
No (DASS-A ≤ 7)	Ref		Ref		Ref	Ref	Ref	
Yes (DASS-A > 7)	0.98 (0.76–1.23)		0.99 (0.78–1.26)		1.38 (1.09–1.74) **	1.44 (1.04–1.99) *	1.05 (0.80–1.39)	
Stress		-		-				-
No (DASS-S ≤ 14)	Ref		Ref		Ref		Ref	
Yes (DASS-S > 14)	1.08 (0.78–1.52)		0.83 (0.59–1.17)		1.12 (0.79–1.59)		0.90 (0.60–1.34)	

Values are odds ratios (ORs) and adjusted odds ratios (aORs) with 95% confidence intervals (CIs) from univariable and multivariable logistic regression models, respectively. Ref = reference category. Age group, sex and marital status were entered into every adjusted model. Each remaining covariate entered a given outcome’s model only where its univariable association with that outcome reached *p* < 0.20, so the adjustment set differs between outcomes and covariates that did not enter are shown as a hyphen (-). Screening *p*-values and the adjustment set for each outcome are given in Supplementary Table S3, the same models with study site added in Supplementary Table S2, adjusted prevalence ratios from modified Poisson models with robust variance in Supplementary Table S4, and model diagnostics in Supplementary Table S13. BMI-defined overweight/obesity: BMI ≥ 25 kg/m^2^. Central obesity: WC ≥ 94 cm for men and ≥80 cm for women; WHR ≥ 0.90 for men and ≥0.85 for women; WHtR ≥ 0.50. * *p* < 0.05, ** *p* < 0.01, and *** *p* < 0.001. Abbreviations: BMI, body mass index; WC, waist circumference; WHR, waist-to-hip ratio; WHtR, waist-to-height ratio; WHO, World Health Organization.

**Table 3:** Factors associated with BMI-WHtR phenotypes: results of univariable and multivariable multinomial logistic regression analyses (n = 1356). Reference category: Neither BMI-defined overweight/obesity nor central obesity (BMI < 25 kg/m^2^ & WHtR < 0.50), n = 209 (15.4%).

Variables	BMI-Defined Overweight/Obesity Only (n = 61, 4.5%)		Central Obesity Only (n = 322, 23.7%)		Combined (n = 764, 56.3%)	
	OR (95% CI)	aOR (95% CI)	OR (95% CI)	aOR (95% CI)	OR (95% CI)	aOR (95% CI)
**Socio-demographics**						
Age group (years)						
18–39	Ref	Ref	Ref	Ref	Ref	Ref
40–59	2.41 (1.18–4.95) *	1.65 (0.77–3.52)	3.04 (1.98–4.64) ***	2.24 (1.42–3.54) ***	5.91 (3.98–8.78) ***	4.09 (2.65–6.33) ***
60–85	2.73 (1.30–5.72) **	1.82 (0.84–3.97)	2.60 (1.65–4.10) ***	2.03 (1.25–3.31) **	7.19 (4.76–10.88) ***	4.94 (3.14–7.78) ***
Sex						
Male	Ref	Ref	Ref	Ref	Ref	Ref
Female	2.17 (1.22–3.87) **	2.21 (1.23–3.99) **	2.48 (1.73–3.54) ***	2.40 (1.66–3.47) ***	4.20 (3.05–5.79) ***	4.04 (2.87–5.68) ***
Marital status						
Never-married	Ref	Ref	Ref	Ref	Ref	Ref
Ever-married	4.72 (1.41–15.82) *	3.85 (1.08–13.72) *	3.69 (2.09–6.50) ***	2.63 (1.41–4.89) **	4.94 (3.06–7.96) ***	2.59 (1.48–4.55) ***
Education level		-		-		-
Pre-primary	Ref		Ref		Ref	
Primary	0.42 (0.16–1.12)		0.61 (0.31–1.20)		0.77 (0.41–1.43)	
Secondary	0.56 (0.21–1.48)		0.60 (0.31–1.19)		0.69 (0.37–1.28)	
Tertiary	0.35 (0.09–1.39)		0.64 (0.28–1.48)		0.93 (0.44–1.97)	
Monthly income (MWK)		-		-		-
≤150,000	Ref		Ref		Ref	
>150,000	1.02 (0.54–1.90)		1.17 (0.80–1.71)		1.12 (0.80–1.56)	
**Clinical characteristics**						
Duration of diabetes (years)						
<2	Ref	-	Ref	-	Ref	-
≥2	1.59 (0.73–3.46)		1.36 (0.88–2.12)		1.36 (0.93–2.00)	
Family history of diabetes						
No/do not know	Ref	-	Ref	-	Ref	-
Yes	1.54 (0.87–2.73)		1.06 (0.74–1.51)		1.19 (0.87–1.62)	
Current treatment		-		-		-
Lifestyle modification	Ref		Ref		Ref	
Medication	0.28 (0.06–1.43)		0.51 (0.14–1.89)		0.64 (0.19–2.21)	
**Behavioral factors**						
Total meal frequency per week						
<20 meals/week	Ref	Ref	Ref	Ref	Ref	Ref
20 meals/week	6.58 (1.33–32.47) *	7.62 (1.47–39.66) *	1.52 (0.39–5.90)	1.78 (0.43–7.32)	3.40 (1.01–11.44) *	3.88 (1.04–14.49) *
21 meals/week	1.57 (0.82–3.02)	1.56 (0.80–3.04)	0.79 (0.55–1.14)	0.80 (0.54–1.16)	1.27 (0.91–1.75)	1.25 (0.87–1.78)
Late-evening eating behavior		-		-		-
No	Ref		Ref		Ref	
Yes	0.86 (0.48–1.53)		1.36 (0.96–1.93)		1.29 (0.95–1.75)	
Physical Activity (WHO Recommendation: ≥ 600 MET-min/week)						
Yes	Ref	Ref	Ref	Ref	Ref	Ref
No	1.84 (0.78–4.33)	1.71 (0.71–4.10)	1.05 (0.57–1.94)	1.01 (0.53–1.92)	2.26 (1.35–3.79) **	1.97 (1.13–3.45) *
Sedentary behavior (hrs/day)						
<8	Ref	-	Ref	-	Ref	-
≥8	1.03 (0.40–2.69)		0.83 (0.45–1.53)		0.86 (0.51–1.46)	
Mental Health (DASS-21)						
Depression		-		-		-
No (DASS-D ≤ 9)	Ref		Ref		Ref	
Yes (DASS-D > 9)	0.72 (0.37–1.41)		0.96 (0.65–1.40)		0.87 (0.62–1.22)	
Anxiety		-		-		-
No (DASS-A ≤ 7)	Ref		Ref		Ref	
Yes (DASS-A > 7)	0.53 (0.28–1.00)		0.87 (0.61–1.25)		0.94 (0.69–1.29)	
Stress		-		-		-
No (DASS-S ≤ 14)	Ref		Ref		Ref	
Yes (DASS-S > 14)	1.22 (0.54–2.76)		0.86 (0.50–1.47)		0.98 (0.61–1.55)	

Values are odds ratios (ORs) and adjusted odds ratios (aORs) with 95% confidence intervals (CIs) from univariable and multivariable multinominal logistic regression, respectively, relative to the reference category (neither BMI-defined overweight/obesity nor central obesity). Group sizes and proportions are given in the column headers. Age group, sex and marital status were entered into the adjusted model; each remaining covariate entered only where its univariable association with the phenotype outcome reached *p* < 0.10, and covariates that did not enter are shown as a hyphen (-). Screening *p*-values are given in Supplementary Table S3, and the same model with study site added in Supplementary Table S2. BMI-defined overweight/obesity: BMI ≥ 25 kg/m^2^, central obesity: WHtR ≥ 0.50. * *p* < 0.05, ** *p* < 0.01, and *** *p* < 0.001. Estimates for the BMI-defined overweight/obesity-only phenotype (n = 61), and particularly the 20 meals/week contrast (n = 46), are imprecise because of sparse cell counts and should be regarded as hypothesis-generating; observation counts for every predictor category within each phenotype are given in Supplementary Table S7, and penalized binary-contrast estimates, which do not materially stabilize this contrast, in Supplementary Table S8. Abbreviations: BMI, body mass index; WHtR, waist-to-height ratio; MET, metabolic equivalent of task; DASS-21, Depression Anxiety Stress Scales-21; WHO, World Health Organization; OR, odds ratio; aOR, adjusted odds ratio; CI, confidence interval.

## Data Availability

The datasets used during the current study are available from the corresponding author on reasonable request.

## Supplementary Materials

The following supporting information can be downloaded at https://www.mdpi.com/article/10.3390/diseases14090334/s1, Table S1: Prevalence of each adiposity definition and distribution of BMI-WHtR phenotypes by study site; Table S2: Sensitivity analysis of adjusted estimates from the models reported in Table 2 and Table 3 before and after adding study site as a fixed-effect covariate; Table S3: Univariable screening p-values for every candidate covariate and the resulting adjustment set for each outcome; Table S4: Adjusted odds ratios compared with adjusted prevalence ratios from modified Poisson regression with robust variance for each outcome’s adjustment set; Table S5: Adjusted predicted prevalence of each adiposity definition by principal covariate; Table S6: Sensitivity of WHtR-defined central obesity prevalence and BMI-WHtR phenotype distribution to the choice of WHtR threshold; Table S7: Number of observations in each predictor category within each BMI-WHtR phenotype; Table S8: BMI-WHtR phenotype: maximum-likelihood multinomial estimates compared with Firth-penalized estimates of the corresponding binary contrasts (sensitivity analysis); Table S9: Sensitivity of the meal-frequency association to the categorization of weekly meal frequency; Table S10: Adiposity among female participants aged <50 and ≥50 years; Table S11: Prevalence of each adiposity definition by participant characteristics, with unadjusted omnibus tests; Table S12: Completeness of every variable used in the analysis; Table S13: Model diagnostics and goodness-of-fit for the four binary logistic models; Table S14: STROBE statement-checklist of items that should be included in reports of cross-sectional studies.
