# Supplementary material for: Discordance Between BMI-Defined Overweight/Obesity and Waist-to-Height Ratio-Defined Central Obesity Among Adults with Type 2 Diabetes in Malawi: A Multicenter Cross-Sectional Study
Source: Diseases. 2026 Sep 13;14(9):334. doi: 10.3390/diseases14090334 (PMC13605006; doi:10.3390/diseases14090334)
Supplement: Supplementary file 1 [file diseases-14-00334-s001.zip › diseases-4503694-supplementary.pdf]

Supplementary Material

Discordance Between BMI-Defined Overweight/Obesity and Waist-to-Height Ratio-Defined Central Obesity Among Adults with Type 2 Diabetes in Malawi: A Multicenter Cross-Sectional Study

Table S1. Prevalence of each adiposity definition and distribution of BMI-WHtR phenotypes, by study site

**Measure**	**Mzuzu Central Hospital, n=202**	**Kamuzu Central Hospital, n=599**	**Zomba Central Hospital, n=555**	χ^2	**p-value**
BMI-defined overweight/obesity (BMI ≥25 kg/m^2)	135 (66.8)	326 (54.4)	364 (65.6)	18.64	<0.001
Central obesity, WC (≥94 cm men / ≥80 cm women)	142 (70.3)	367 (61.3)	406 (73.2)	19.40	<0.001
Central obesity, WHR (≥0.90 men / ≥0.85 women)	131 (64.9)	363 (60.6)	391 (70.5)	12.35	0.002
Central obesity, WHtR (≥0.50)	167 (82.7)	429 (71.6)	490 (88.3)	51.19	<0.001
BMI-WHtR phenotype: Neither	32 (15.8)	125 (20.9)	52 (9.4)		
BMI-WHtR phenotype: BMI-only	3 (1.5)	45 (7.5)	13 (2.3)		
BMI-WHtR phenotype: Central-only	35 (17.3)	148 (24.7)	139 (25.0)		
BMI-WHtR phenotype: Combined	132 (65.3)	281 (46.9)	351 (63.2)		
Phenotype distribution (overall test)				67.84 (df=6)	<0.001
Discordant phenotypes combined (BMI-only + central-only)	38 (18.8)	193 (32.2)	152 (27.4)		

Values are n (%) within each site. χ^2 tests compare the three sites. The phenotype rows are tested jointly by a single 3×4 χ^2 test. Discordance (BMI-only plus central-obesity-only) was present at all three hospitals.

Table S2. Sensitivity analysis: adjusted estimates from the models reported in Tables 2 and 3, before and after adding study site as a fixed-effect covariate

Characteristic	Primary Model, aOR (95% CI)	With Study Site Added, aOR (95% CI)	
BMI-defined overweight/obesity (BMI ≥25 kg/m²)			
Age group			
18-39 years (reference)	1.00	1.00	
40-59 years	2.30 (1.66-3.18) ***	2.24 (1.62-3.11) ***	
60-85 years	2.95 (2.11-4.13) ***	2.95 (2.10-4.13) ***	
Sex			
Male (reference)	1.00	1.00	
Female	2.19 (1.72-2.79) ***	2.20 (1.73-2.81) ***	
Marital status			
Never-married (reference)	1.00	1.00	
Ever-married	1.65 (1.03-2.64) *	1.66 (1.04-2.65) *	
Education level			
Pre-primary (reference)	1.00	1.00	
Primary	-	-	
Secondary	-	-	
Tertiary	-	-	
Monthly income			
≤150,000 MWK (reference)	1.00	1.00	
>150,000 MWK	-	-	
Diabetes duration			
<2 years (reference)	1.00	1.00	
≥2 years	-	-	
Family history of diabetes			
No / do not know (reference)	1.00	1.00	
Yes	1.02 (0.81-1.29)	1.00 (0.79-1.27)	
Current treatment			

Lifestyle modification (reference)	1.00	1.00	
Medication	-	-	
Meal frequency			
<20 meals/week (reference)	1.00	1.00	
21 meals/week	1.47 (1.15-1.89) **	1.52 (1.19-1.95) ***	
20 meals/week	2.72 (1.29-5.73) **	2.65 (1.24-5.67) *	
Late-evening eating behavior			
No (reference)	1.00	1.00	
Yes	-	-	
Physical activity			
Meets WHO recommendation (reference)	1.00	1.00	
Does not meet	1.93 (1.34-2.78) ***	1.89 (1.31-2.73) ***	
Sedentary behavior			
<8 hours/day (reference)	1.00	1.00	
≥8 hours/day	-	-	
Depression			
Normal (reference)	1.00	1.00	
Depressive symptoms	-	-	
Anxiety			
Normal (reference)	1.00	1.00	
Anxiety symptoms	-	-	
Stress			
Normal (reference)	1.00	1.00	
Stress symptoms	-	-	
Study site			
Mzuzu Central Hospital (reference)	1.00	1.00	
Kamuzu Central Hospital	-	0.67 (0.47-0.95) *	
Zomba Central Hospital	-	1.10 (0.76-1.58)	

Central obesity, WC (≥94 cm men / ≥80 cm women)			
Age group			
18-39 years (reference)	1.00	1.00	
40-59 years	2.76 (1.90-4.01) ***	2.70 (1.85-3.92) ***	
60-85 years	2.67 (1.82-3.93) ***	2.69 (1.83-3.96) ***	
Sex			
Male (reference)	1.00	1.00	
Female	10.16 (7.67-13.45) ***	10.50 (7.90-13.95) ***	
Marital status			
Never-married (reference)	1.00	1.00	
Ever-married	1.96 (1.16-3.32) *	1.99 (1.17-3.38) *	
Education level			
Pre-primary (reference)	1.00	1.00	
Primary	0.72 (0.42-1.22)	0.84 (0.49-1.46)	
Secondary	0.99 (0.58-1.71)	1.12 (0.64-1.94)	
Tertiary	1.37 (0.72-2.58)	1.55 (0.81-2.96)	
Monthly income			
≤150,000 MWK (reference)	1.00	1.00	
>150,000 MWK	-	-	
Diabetes duration			
<2 years (reference)	1.00	1.00	
≥2 years	-	-	
Family history of diabetes			
No / do not know (reference)	1.00	1.00	
Yes	0.94 (0.71-1.24)	0.92 (0.70-1.22)	
Current treatment			
Lifestyle modification (reference)	1.00	1.00	
Medication	-	-	
Meal frequency			
<20 meals/week (reference)	1.00	1.00	

21 meals/week	-	-	
20 meals/week	-	-	
Late-evening eating behavior			
No (reference)	1.00	1.00	
Yes	-	-	
Physical activity			
Meets WHO recommendation (reference)	1.00	1.00	
Does not meet	1.33 (0.89-1.99)	1.32 (0.87-1.98)	
Sedentary behavior			
<8 hours/day (reference)	1.00	1.00	
≥8 hours/day	-	-	
Depression			
Normal (reference)	1.00	1.00	
Depressive symptoms	-	-	
Anxiety			
Normal (reference)	1.00	1.00	
Anxiety symptoms	-	-	
Stress			
Normal (reference)	1.00	1.00	
Stress symptoms	-	-	
Study site			
Mzuzu Central Hospital (reference)	1.00	1.00	
Kamuzu Central Hospital	-	0.74 (0.50-1.11)	
Zomba Central Hospital	-	1.41 (0.93-2.15)	
Central obesity, WHR (≥0.90 men / ≥0.85 women)			
Age group			
18-39 years (reference)	1.00	1.00	
40-59 years	1.66 (1.20-2.30) **	1.64 (1.19-2.27) **	

60-85 years	2.11 (1.51-2.94) ***	2.12 (1.51-2.96) ***	
Sex			
Male (reference)	1.00	1.00	
Female	1.92 (1.51-2.44) ***	1.94 (1.52-2.46) ***	
Marital status			
Never-married (reference)	1.00	1.00	
Ever-married	1.24 (0.78-1.95)	1.23 (0.77-1.94)	
Education level			
Pre-primary (reference)	1.00	1.00	
Primary	0.74 (0.47-1.16)	0.83 (0.52-1.31)	
Secondary	0.71 (0.45-1.12)	0.78 (0.49-1.24)	
Tertiary	0.82 (0.47-1.42)	0.89 (0.51-1.55)	
Monthly income			
≤150,000 MWK (reference)	1.00	1.00	
>150,000 MWK	-	-	
Diabetes duration			
<2 years (reference)	1.00	1.00	
≥2 years	-	-	
Family history of diabetes			
No / do not know (reference)	1.00	1.00	
Yes	-	-	
Current treatment			
Lifestyle modification (reference)	1.00	1.00	
Medication	1.55 (0.74-3.23)	1.51 (0.72-3.16)	
Meal frequency			
<20 meals/week (reference)	1.00	1.00	
21 meals/week	-	-	
20 meals/week	-	-	
Late-evening eating behavior			
No (reference)	1.00	1.00	

Yes	-	-	
Physical activity			
Meets WHO recommendation (reference)	1.00	1.00	
Does not meet	-	-	
Sedentary behavior			
<8 hours/day (reference)	1.00	1.00	
≥8 hours/day	-	-	
Depression			
Normal (reference)	1.00	1.00	
Depressive symptoms	0.94 (0.66-1.33)	0.89 (0.63-1.27)	
Anxiety			
Normal (reference)	1.00	1.00	
Anxiety symptoms	1.44 (1.04-1.99) *	1.41 (1.02-1.96) *	
Stress			
Normal (reference)	1.00	1.00	
Stress symptoms	-	-	
Study site			
Mzuzu Central Hospital (reference)	1.00	1.00	
Kamuzu Central Hospital	-	0.88 (0.63-1.24)	
Zomba Central Hospital	-	1.31 (0.91-1.87)	
Central obesity, WHtR (≥0.50)			
Age group			
18-39 years (reference)	1.00	1.00	
40-59 years	2.85 (1.98-4.09) ***	2.87 (1.98-4.15) ***	
60-85 years	3.07 (2.11-4.47) ***	3.21 (2.18-4.73) ***	
Sex			
Male (reference)	1.00	1.00	
Female	2.77 (2.08-3.68) ***	2.85 (2.13-3.82) ***	

Marital status			
Never-married (reference)	1.00	1.00	
Ever-married	2.12 (1.31-3.43) **	2.23 (1.37-3.64) **	
Education level			
Pre-primary (reference)	1.00	1.00	
Primary	-	-	
Secondary	-	-	
Tertiary	-	-	
Monthly income			
≤150,000 MWK (reference)	1.00	1.00	
>150,000 MWK	-	-	
Diabetes duration			
<2 years (reference)	1.00	1.00	
≥2 years	-	-	
Family history of diabetes			
No / do not know (reference)	1.00	1.00	
Yes	-	-	
Current treatment			
Lifestyle modification (reference)	1.00	1.00	
Medication	-	-	
Meal frequency			
<20 meals/week (reference)	1.00	1.00	
21 meals/week	-	-	
20 meals/week	-	-	
Late-evening eating behavior			
No (reference)	1.00	1.00	
Yes	1.36 (1.02-1.80) *	1.12 (0.83-1.51)	
Physical activity			
Meets WHO recommendation (reference)	1.00	1.00	

Does not meet	1.43 (0.90-2.25)	1.37 (0.86-2.17)	
Sedentary behavior			
<8 hours/day (reference)	1.00	1.00	
≥8 hours/day	-	-	
Depression			
Normal (reference)	1.00	1.00	
Depressive symptoms	-	-	
Anxiety			
Normal (reference)	1.00	1.00	
Anxiety symptoms	-	-	
Stress			
Normal (reference)	1.00	1.00	
Stress symptoms	-	-	
Study site			
Mzuzu Central Hospital (reference)	1.00	1.00	
Kamuzu Central Hospital	-	0.60 (0.39-0.92) *	
Zomba Central Hospital	-	1.90 (1.17-3.07) **	
Phenotype: BMI-only vs neither			
Age group			
18-39 years (reference)	1.00	1.00	
40-59 years	1.65 (0.77-3.52)	1.79 (0.85-3.81)	
60-85 years	1.82 (0.84-3.97)	1.91 (0.88-4.18)	
Sex			
Male (reference)	1.00	1.00	
Female	2.21 (1.23-3.99) **	2.28 (1.26-4.13) **	
Marital status			
Never-married (reference)	1.00	1.00	
Ever-married	3.85 (1.08-13.72) *	3.98 (1.13-14.07) *	
Education level			

Pre-primary (reference)	1.00	1.00	
Primary	-	-	
Secondary	-	-	
Tertiary	-	-	
Monthly income			
≤150,000 MWK (reference)	1.00	1.00	
>150,000 MWK	-	-	
Diabetes duration			
<2 years (reference)	1.00	1.00	
≥2 years	-	-	
Family history of diabetes			
No / do not know (reference)	1.00	1.00	
Yes	-	-	
Current treatment			
Lifestyle modification (reference)	1.00	1.00	
Medication	-	-	
Meal frequency			
<20 meals/week (reference)	1.00	1.00	
21 meals/week	1.56 (0.80-3.04)	1.60 (0.82-3.13)	
20 meals/week	7.62 (1.47-39.66) *	8.30 (1.58-43.56) *	
Late-evening eating behavior			
No (reference)	1.00	1.00	
Yes	-	-	
Physical activity			
Meets WHO recommendation (reference)	1.00	1.00	
Does not meet	1.71 (0.71-4.10)	1.89 (0.78-4.60)	
Sedentary behavior			
<8 hours/day (reference)	1.00	1.00	
≥8 hours/day	-	-	

Depression			
Normal (reference)	1.00	1.00	
Depressive symptoms	-	-	
Anxiety			
Normal (reference)	1.00	1.00	
Anxiety symptoms	-	-	
Stress			
Normal (reference)	1.00	1.00	
Stress symptoms	-	-	
Study site			
Mzuzu Central Hospital (reference)	1.00	1.00	
Kamuzu Central Hospital	-	4.31 (1.24-15.01) *	
Zomba Central Hospital	-	3.29 (0.85-12.73)	
Phenotype: Central-only vs neither			
Age group			
18-39 years (reference)	1.00	1.00	
40-59 years	2.24 (1.42-3.54) ***	2.30 (1.45-3.64) ***	
60-85 years	2.03 (1.25-3.31) **	2.14 (1.31-3.50) **	
Sex			
Male (reference)	1.00	1.00	
Female	2.40 (1.66-3.47) ***	2.53 (1.74-3.67) ***	
Marital status			
Never-married (reference)	1.00	1.00	
Ever-married	2.63 (1.41-4.89) **	2.81 (1.50-5.26) **	
Education level			
Pre-primary (reference)	1.00	1.00	
Primary	-	-	
Secondary	-	-	
Tertiary	-	-	

Monthly income			
≤150,000 MWK (reference)	1.00	1.00	
>150,000 MWK	-	-	
Diabetes duration			
<2 years (reference)	1.00	1.00	
≥2 years	-	-	
Family history of diabetes			
No / do not know (reference)	1.00	1.00	
Yes	-	-	
Current treatment			
Lifestyle modification (reference)	1.00	1.00	
Medication	-	-	
Meal frequency			
<20 meals/week (reference)	1.00	1.00	
21 meals/week	0.80 (0.54-1.16)	0.88 (0.60-1.29)	
20 meals/week	1.78 (0.43-7.32)	1.78 (0.42-7.59)	
Late-evening eating behavior			
No (reference)	1.00	1.00	
Yes	-	-	
Physical activity			
Meets WHO recommendation (reference)	1.00	1.00	
Does not meet	1.01 (0.53-1.92)	1.04 (0.55-1.99)	
Sedentary behavior			
<8 hours/day (reference)	1.00	1.00	
≥8 hours/day	-	-	
Depression			
Normal (reference)	1.00	1.00	
Depressive symptoms	-	-	
Anxiety			

Normal (reference)	1.00	1.00	
Anxiety symptoms	-	-	
Stress			
Normal (reference)	1.00	1.00	
Stress symptoms	-	-	
Study site			
Mzuzu Central Hospital (reference)	1.00	1.00	
Kamuzu Central Hospital	-	1.19 (0.68-2.08)	
Zomba Central Hospital	-	2.91 (1.59-5.32) ***	
Phenotype: Combined vs neither			
Age group			
18-39 years (reference)	1.00	1.00	
40-59 years	4.09 (2.65-6.33) ***	4.10 (2.64-6.38) ***	
60-85 years	4.94 (3.14-7.78) ***	5.19 (3.27-8.24) ***	
Sex			
Male (reference)	1.00	1.00	
Female	4.04 (2.87-5.68) ***	4.25 (3.00-6.02) ***	
Marital status			
Never-married (reference)	1.00	1.00	
Ever-married	2.59 (1.48-4.55) ***	2.74 (1.55-4.86) ***	
Education level			
Pre-primary (reference)	1.00	1.00	
Primary	-	-	
Secondary	-	-	
Tertiary	-	-	
Monthly income			
≤150,000 MWK (reference)	1.00	1.00	
>150,000 MWK	-	-	
Diabetes duration			

<2 years (reference)	1.00	1.00	
≥2 years	-	-	
Family history of diabetes			
No / do not know (reference)	1.00	1.00	
Yes	-	-	
Current treatment			
Lifestyle modification (reference)	1.00	1.00	
Medication	-	-	
Meal frequency			
<20 meals/week (reference)	1.00	1.00	
21 meals/week	1.25 (0.87-1.78)	1.38 (0.96-1.99)	
20 meals/week	3.88 (1.04-14.49) *	3.78 (0.96-14.80)	
Late-evening eating behavior			
No (reference)	1.00	1.00	
Yes	-	-	
Physical activity			
Meets WHO recommendation (reference)	1.00	1.00	
Does not meet	1.97 (1.13-3.45) *	1.98 (1.12-3.49) *	
Sedentary behavior			
<8 hours/day (reference)	1.00	1.00	
≥8 hours/day	-	-	
Depression			
Normal (reference)	1.00	1.00	
Depressive symptoms	-	-	
Anxiety			
Normal (reference)	1.00	1.00	
Anxiety symptoms	-	-	
Stress			
Normal (reference)	1.00	1.00	

Stress symptoms	-	-	
Study site			
Mzuzu Central Hospital (reference)	1.00	1.00	
Kamuzu Central Hospital	-	0.64 (0.40-1.04)	
Zomba Central Hospital	-	2.23 (1.31-3.80) **	

The primary models are exactly those reported in Tables 2 and 3 of the manuscript. The second column repeats each model with study site entered as an additional fixed-effect covariate, holding the covariate set otherwise unchanged. A dash indicates a covariate that did not meet the univariable entry criterion for that outcome and was therefore not in the model (Table S3). The demographic associations are materially unchanged by site adjustment. Two associations do not survive this: late-evening eating behavior and WHtR-defined central obesity is attenuated to the null (aOR 1.36, 95% CI 1.02-1.80 in the primary model; 1.12, 95% CI 0.83-1.51 with site), and the 20 meals/week contrast for the combined phenotype crosses the null (3.88, 95% CI 1.04-14.49 in the primary model; 3.78, 95% CI 0.96-14.80 with site). Study site is itself associated with WHtR-defined central obesity (Zomba vs Mzuzu aOR 1.90, 95% CI 1.17-3.07) and with the central-obesity-only and combined phenotypes. Two indicator variables for three sites are estimable without difficulty; these models are reported alongside the primary specification rather than as a replacement for it because study site is a feature of the recruitment design rather than a participant-level characteristic. What three of the clusters would not support is cluster-robust variance estimation, which was not used.

Table S3. Univariable screening p-values for every candidate covariate, and the resulting adjustment set for each outcome

Candidate Covariate	BMI-Defined Overweight/Obesity	Central Obesity (WC)	Central Obesity (WHR)	Central Obesity (WHtR)	BMI-WHtR Phenotype
Age group	Forced	Forced	Forced	Forced	Forced
Sex	Forced	Forced	Forced	Forced	Forced
Marital status	Forced	Forced	Forced	Forced	Forced
Education level	0.730	0.019 (retained)	0.023 (retained)	0.470	0.522
Monthly income	0.959	0.687	0.250	0.407	0.848
Diabetes duration	0.335	0.849	0.549	0.215	0.397
Family history of diabetes	0.168 (retained)	0.033 (retained)	0.238	0.775	0.400
Current treatment	0.845	0.586	0.153 (retained)	0.867	0.469
Meal frequency	<0.001 (retained)	0.395	0.642	0.704	0.004 (retained)

Late-evening eating behavior	0.729	0.440	0.204	0.026 (retained)	0.145
Physical activity	<0.001 (retained)	0.030 (retained)	0.510	0.028 (retained)	<0.001 (retained)
Sedentary behavior	0.909	0.955	0.583	0.486	0.915
Depression	0.324	0.226	0.163 (retained)	0.792	0.708
Anxiety	0.897	0.938	0.008 (retained)	0.716	0.212
Stress	0.627	0.289	0.524	0.598	0.832

Age group, sex and marital status were entered into every model as core demographic confounders specified in advance. Every other candidate entered a given outcome's adjusted model only where its univariable association with that outcome reached p<0.20; for the multinomial phenotype outcome a stricter threshold of p<0.10 was applied, because the BMI-only stratum contains only 61 participants and a larger adjustment set would be poorly identified against it. p-values are likelihood-ratio tests against the null model, so a single omnibus p-value is reported for covariates with more than two levels. Because the screen is applied to each outcome separately, the adjustment set differs across outcomes, and covariates that did not enter a given model are shown as a dash in Tables 2 and 3. No stepwise, forward or backward procedure was used at any stage: each adjusted model is a single multivariable fit of the three forced covariates plus those that passed the screen. Study site is not among the covariates screened; Table S2 reports every model with and without site adjustment.

Table S4. Adjusted odds ratios compared with adjusted prevalence ratios from modified Poisson regression with robust variance, for each outcome's adjustment set

**Characteristic**	**aOR (95% CI)**	**p**	**aPR (95% CI)**	**p**
BMI-defined overweight/obesity				
Age group				
18-39 years (reference)	1.00		1.00	
40-59 years	2.30 (1.66-3.18)	<0.001	1.51 (1.27-1.78)	<0.001
60-85 years	2.95 (2.11-4.13)	<0.001	1.63 (1.37-1.92)	<0.001
Sex				
Male (reference)	1.00		1.00	
Female	2.19 (1.72-2.79)	<0.001	1.36 (1.23-1.50)	<0.001
Marital status				
Never-married (reference)	1.00		1.00	
Ever-married	1.65 (1.03-2.64)	0.035	1.26 (0.99-1.61)	0.062

Family history of diabetes				
No / do not know (reference)	1.00		1.00	
Yes	1.02 (0.81-1.29)	0.864	1.01 (0.93-1.09)	0.875
Meal frequency				
<20 meals/week (reference)	1.00		1.00	
21 meals/week	1.47 (1.15-1.89)	0.002	1.16 (1.05-1.28)	0.002
20 meals/week	2.72 (1.29-5.73)	0.008	1.35 (1.14-1.61)	<0.001
Physical activity				
Meets WHO recommendation (reference)	1.00		1.00	
Does not meet	1.93 (1.34-2.78)	<0.001	1.21 (1.10-1.33)	<0.001
Central obesity (WC)				
Age group				
18-39 years (reference)	1.00		1.00	
40-59 years	2.76 (1.90-4.01)	<0.001	1.40 (1.23-1.58)	<0.001
60-85 years	2.67 (1.82-3.93)	<0.001	1.39 (1.22-1.57)	<0.001
Sex				
Male (reference)	1.00		1.00	
Female	10.16 (7.67-13.45)	<0.001	2.25 (2.01-2.53)	<0.001
Marital status				
Never-married (reference)	1.00		1.00	
Ever-married	1.96 (1.16-3.32)	0.012	1.24 (1.03-1.48)	0.021
Education level				
Pre-primary (reference)	1.00		1.00	
Primary	0.72 (0.42-1.22)	0.222	0.94 (0.84-1.04)	0.206
Secondary	0.99 (0.58-1.71)	0.980	1.02 (0.92-1.14)	0.706
Tertiary	1.37 (0.72-2.58)	0.335	1.15 (1.00-1.32)	0.059
Family history of diabetes				
No / do not know (reference)	1.00		1.00	
Yes	0.94 (0.71-1.24)	0.654	0.98 (0.92-1.05)	0.550
Physical activity				

Meets WHO recommendation (reference)	1.00		1.00	
Does not meet	1.33 (0.89-1.99)	0.169	1.06 (0.98-1.16)	0.167
Central obesity (WHR)				
Age group				
18-39 years (reference)	1.00		1.00	
40-59 years	1.66 (1.20-2.30)	0.002	1.23 (1.08-1.41)	0.002
60-85 years	2.11 (1.51-2.94)	<0.001	1.33 (1.16-1.52)	<0.001
Sex				
Male (reference)	1.00		1.00	
Female	1.92 (1.51-2.44)	<0.001	1.27 (1.15-1.39)	<0.001
Marital status				
Never-married (reference)	1.00		1.00	
Ever-married	1.24 (0.78-1.95)	0.363	1.09 (0.90-1.31)	0.384
Education level				
Pre-primary (reference)	1.00		1.00	
Primary	0.74 (0.47-1.16)	0.190	0.92 (0.82-1.04)	0.176
Secondary	0.71 (0.45-1.12)	0.145	0.91 (0.80-1.03)	0.151
Tertiary	0.82 (0.47-1.42)	0.473	0.96 (0.82-1.13)	0.650
Current treatment				
Lifestyle modification (reference)	1.00		1.00	
Medication	1.55 (0.74-3.23)	0.247	1.18 (0.85-1.64)	0.311
Depression				
Normal (reference)	1.00		1.00	
Depressive symptoms	0.94 (0.66-1.33)	0.725	0.98 (0.88-1.09)	0.679
Anxiety				
Normal (reference)	1.00		1.00	
Anxiety symptoms	1.44 (1.04-1.99)	0.028	1.12 (1.02-1.24)	0.020
Central obesity (WHtR)				
Age group				
18-39 years (reference)	1.00		1.00	

40-59 years	2.85 (1.98-4.09)	<0.001	1.31 (1.19-1.46)	<0.001
60-85 years	3.07 (2.11-4.47)	<0.001	1.33 (1.20-1.47)	<0.001
Sex				
Male (reference)	1.00		1.00	
Female	2.77 (2.08-3.68)	<0.001	1.23 (1.15-1.31)	<0.001
Marital status				
Never-married (reference)	1.00		1.00	
Ever-married	2.12 (1.31-3.43)	0.002	1.25 (1.05-1.49)	0.010
Late-evening eating behavior				
No (reference)	1.00		1.00	
Yes	1.36 (1.02-1.80)	0.036	1.06 (1.00-1.11)	0.035
Physical activity				
Meets WHO recommendation (reference)	1.00		1.00	
Does not meet	1.43 (0.90-2.25)	0.127	1.05 (0.99-1.12)	0.098

aPR, adjusted prevalence ratio. Prevalence ratios were estimated by Poisson regression with a robust (sandwich, HC0) variance estimator on the same covariate set. Because all four outcomes are common (prevalence 60.8-80.1%), the odds ratios are systematically further from the null than the corresponding prevalence ratios; the direction and statistical significance of the principal associations are unchanged. Each panel contains the covariates retained for that outcome under the screening rule set out in Table S2.

Table S5. Adjusted predicted prevalence (%) of each adiposity definition, by principal covariate

Characteristic	Category	BMI-Defined Overweight/Obesity	Central Obesity (WC)	Central Obesity (WHR)	Central Obesity (WHtR)
Age group	18-39 years	43.9	54.0	54.3	66.2
	40-59 years	62.4	70.9	65.6	83.4
	60-85 years	68.2	70.9	70.9	84.8
Sex	Male	49.8	37.7	56.0	70.6
	Female	67.2	84.3	70.7	85.9
Marital status	Never-married	50.6	56.9	61.2	68.5
	Ever-married	61.7	68.3	65.6	81.2

Characteristic	Category	BMI-Defined Overweight/Obesity	Central Obesity (WC)	Central Obesity (WHR)	Central Obesity (WHtR)
Study site	Mzuzu Central Hospital	63.9	67.5	64.2	80.7
	Kamuzu Central Hospital	55.3	62.6	61.5	72.8
	Zomba Central Hospital	65.8	72.7	69.8	88.1

Adjusted predicted prevalences were obtained by marginal standardization from the logistic models in Table S2: every participant was assigned in turn to each level of the covariate, holding all other covariates at their observed values, and the predicted probabilities were averaged. Only the covariates common to all models (age, sex and marital status) are shown, because the remaining covariates differ between outcomes. Site-specific adjusted predicted prevalences are not reported here; site-adjusted odds ratios for every model are given in Table S2..

Table S6. Sensitivity of the WHtR-defined central obesity prevalence and of the BMI-WHtR phenotype distribution to the choice of WHtR threshold

**WHtR threshold**	**Central obesity**	**Neither**	**BMI-only**	**Central obesity only**	**Combined**	**Discordant (BMI-only + central only)**	**Observed agreement (%)**	**κ**
≥0.50	1086 (80.1)	209 (15.4)	61 (4.5)	322 (23.7)	764 (56.3)	383 (28.2)	71.8	0.35
≥0.52	991 (73.1)	262 (19.3)	103 (7.6)	269 (19.8)	722 (53.2)	372 (27.4)	72.6	0.39
≥0.53	943 (69.5)	298 (22.0)	115 (8.5)	233 (17.2)	710 (52.4)	348 (25.7)	74.3	0.44
≥0.55	867 (63.9)	338 (24.9)	151 (11.1)	193 (14.2)	674 (49.7)	344 (25.4)	74.6	0.46

Values are n (%) of all 1356 participants. BMI-defined overweight/obesity is held at BMI ≥25 kg/m² throughout. The conventional ≥0.50 threshold is the one used in the main analysis. Discordance between BMI and WHtR remains substantial at every threshold examined, so the central finding does not depend on the cut-off chosen.

Table S7. Number of observations in each predictor category within each BMI-WHtR phenotype

**Characteristic**	**Neither, n=209**	**BMI-Only, n=61**	**Central Obesity only, n=322**	**Combined, n=764**	**Total, n=1356**

Age group					
18-39 years	95	15	73	87	270
40-59 years	63	24	147	341	575
60-85 years	51	22	102	336	511
Sex					
Male	129	26	127	212	494
Female	80	35	195	552	862
Marital status					
Never-married	41	3	20	36	100
Ever-married	168	58	302	728	1256
Education level					
Pre-primary	14	8	34	67	123
Primary	88	21	130	322	561
Secondary	87	28	127	286	528
Tertiary	20	4	31	89	144
Monthly income					
≤150,000 MWK	148	43	217	523	931
>150,000 MWK	61	18	105	241	425
Diabetes duration					
<2 years	45	9	54	128	236
≥2 years	164	52	268	636	1120
Family history of diabetes					
No / do not know	125	30	188	425	768
Yes	84	31	134	339	588
Current treatment					
Lifestyle modification	3	3	9	17	32
Medication	206	58	313	747	1324
Meal frequency					
21 meals/week	132	42	184	508	866
20 meals/week	3	4	8	31	46

<20 meals/week	74	15	130	225	444
Late-evening eating behavior					
No	112	35	148	361	656
Yes	97	26	174	403	700
Physical activity					
Meets WHO recommendation	191	52	293	630	1166
Does not meet	18	9	29	134	190
Sedentary behavior					
<8 hours/day	189	55	296	700	1240
≥8 hours/day	20	6	26	64	116
Depression					
Normal	148	47	231	562	988
Depressive symptoms	61	14	91	202	368
Anxiety					
Normal	129	46	209	482	866
Anxiety symptoms	80	15	113	282	490
Stress					
Normal	183	52	287	671	1193
Stress symptoms	26	9	35	93	163
Study site					
Mzuzu Central Hospital	32	3	35	132	202
Kamuzu Central Hospital	125	45	148	281	599
Zomba Central Hospital	52	13	139	351	555

This table shows the events-per-category position underlying the multinomial model. The BMI-only phenotype contains only 61 participants, and several predictor categories within it contain fewer than 10 observations, which is the source of the wide confidence intervals in Table 3 of the manuscript.

Table S8. BMI-WHtR phenotype: maximum-likelihood multinominal estimates compared with Firth-penalized estimates of the corresponding binary contrasts (sensitivity analysis).

Characteristic	aOR (95% CI)	p	Firth-Penalized aOR (95% CI)	p
BMI-defined overweight/obesity only (n=61) vs neither (n=209)				
Age group				
18-39 years (reference)	1.00		1.00	
40-59 years	1.65 (0.77-3.52)	0.195	1.65 (0.77-3.52)	0.198
60-85 years	1.82 (0.84-3.97)	0.131	1.66 (0.75-3.65)	0.209
Sex				
Male (reference)	1.00		1.00	
Female	2.21 (1.23-3.99)	0.008	2.22 (1.20-4.09)	0.011
Marital status				
Never-married (reference)	1.00		1.00	
Ever-married	3.85 (1.08-13.72)	0.038	3.58 (1.08-11.80)	0.036
Meal frequency				
<20 meals/week (reference)	1.00		1.00	
21 meals/week	1.56 (0.80-3.04)	0.189	1.57 (0.80-3.09)	0.191
20 meals/week	7.62 (1.47-39.66)	0.016	8.79 (1.61-48.03)	0.012
Physical activity				
Meets WHO recommendation (reference)	1.00		1.00	
Does not meet	1.71 (0.71-4.10)	0.233	1.91 (0.78-4.69)	0.159
Central obesity only (n=322) vs neither (n=209)				
Age group				
18-39 years (reference)	1.00		1.00	
40-59 years	2.24 (1.42-3.54)	<0.001	2.21 (1.40-3.51)	<0.001
60-85 years	2.03 (1.25-3.31)	0.004	1.89 (1.15-3.11)	0.012
Sex				
Male (reference)	1.00		1.00	
Female	2.40 (1.66-3.47)	<0.001	2.35 (1.61-3.41)	<0.001
Marital status				
Never-married (reference)	1.00		1.00	

Ever-married	2.63 (1.41-4.89)	0.002	2.51 (1.34-4.72)	0.004
Meal frequency				
<20 meals/week (reference)	1.00		1.00	
21 meals/week	0.80 (0.54-1.16)	0.239	0.84 (0.57-1.23)	0.369
20 meals/week	1.78 (0.43-7.32)	0.426	1.51 (0.38-5.98)	0.555
Physical activity				
Meets WHO recommendation (reference)	1.00		1.00	
Does not meet	1.01 (0.53-1.92)	0.970	0.94 (0.49-1.82)	0.863
Combined phenotype (n=764) vs neither (n=209)				
Age group				
18-39 years (reference)	1.00		1.00	
40-59 years	4.09 (2.65-6.33)	<0.001	3.88 (2.50-6.01)	<0.001
60-85 years	4.94 (3.14-7.78)	<0.001	4.58 (2.89-7.25)	<0.001
Sex				
Male (reference)	1.00		1.00	
Female	4.04 (2.87-5.68)	<0.001	4.25 (2.99-6.04)	<0.001
Marital status				
Never-married (reference)	1.00		1.00	
Ever-married	2.59 (1.48-4.55)	<0.001	2.95 (1.66-5.25)	<0.001
Meal frequency				
<20 meals/week (reference)	1.00		1.00	
21 meals/week	1.25 (0.87-1.78)	0.226	1.27 (0.88-1.84)	0.204
20 meals/week	3.88 (1.04-14.49)	0.043	3.83 (0.97-15.09)	0.055
Physical activity				
Meets WHO recommendation (reference)	1.00		1.00	
Does not meet	1.97 (1.13-3.45)	0.017	1.97 (1.12-3.48)	0.018

Maximum-likelihood estimates come from a single multinomial logistic model with "neither" as the base outcome. Firth-penalized estimates were obtained by fitting the corresponding baseline-category contrast as a penalized (Jeffreys-prior) binary logistic model; confidence intervals are Wald intervals from the penalized information matrix. Because the penalized models are separate binary contrasts rather than a single penalized multinomial fit, they are reported as a sensitivity analysis and are not directly substitutable for the multinomial estimates.

Penalization did not attenuate the principal sparse-data estimate: for the 20 meals/week contrast against the BMI-only phenotype it rose from 7.61 (95% CI 1.46-39.62) to 8.79 (95% CI 1.61-48.03), and the same contrast against the combined phenotype no longer reached significance under penalization (p=0.055 against p=0.043). Direction was unchanged for every association. The meal-frequency contrast for the BMI-only phenotype remains extremely imprecise under both approaches and should be treated as hypothesis-generating.

Table S9. Sensitivity of the meal-frequency association to the categorization of weekly meal frequency

**Outcome**	**20 Meals/Week vs <20 (3-category)**		**21 Meals/Week vs <20 (3-category)**		**Continuous, per +1 Meal/Week**		**<20 vs ≥20 Meals/Week**		**≤20 vs 21 Meals/Week**
	**aOR (95% CI)**	**p**	**aOR (95% CI)**	**p**	**aOR (95% CI)**	**p**	**aOR (95% CI)**	**p**	**aOR (95% CI) / p**
BMI-defined overweight/obesity (BMI ≥25 kg/m²)	2.72 (1.29-5.73)	0.008	1.47 (1.15-1.89)	0.002	1.02 (0.99-1.05)	0.154	0.66 (0.52-0.84)	<0.001	0.74 (0.58-0.94); p=0.013
Central obesity, WC (≥94 cm men / ≥80 cm women)	1.87 (0.80-4.42)	0.151	1.13 (0.84-1.51)	0.417	1.01 (0.97-1.04)	0.670	0.87 (0.65-1.16)	0.331	0.93 (0.70-1.24); p=0.630
Central obesity, WHR (≥0.90 men / ≥0.85 women)	0.68 (0.36-1.29)	0.240	1.01 (0.78-1.30)	0.951	1.00 (0.98-1.03)	0.770	1.01 (0.79-1.30)	0.914	0.96 (0.75-1.22); p=0.719
Central obesity, WHtR (≥0.50)	1.29 (0.52-3.18)	0.579	0.94 (0.69-1.28)	0.700	1.00 (0.96-1.03)	0.902	1.05 (0.77-1.42)	0.764	1.08 (0.80-1.46); p=0.593

Weekly meal frequency is bounded above at 21 (three meals per day for seven days). An amount of 866 participants (63.9%) reported exactly 21 meals, 46 (3.4%) reported 20, and 444 (32.7%) reported fewer than 20, so three approximately equal groups cannot be formed and the categories are not tertiles. All models use the prespecified adjustment set with study site, replacing the categorical meal-frequency term with the specification shown. In the collapsed analyses the higher-frequency group is the reference.

Table S10. Adiposity among female participants aged <50 and ≥50 years

**Outcome**	**Women <50 Years (n=316)**	**Women ≥50 Years (n=546)**	**χ^2**	**p-value**	**aOR (95% CI)**	**p-value**

Central obesity, WC	253/316 (80.1)	478/546 (87.5)	8.12	0.004	1.63 (1.11-2.40)	0.013
Central obesity, WHR	209/316 (66.1)	406/546 (74.4)	6.22	0.013	1.46 (1.07-1.99)	0.016
Central obesity, WHtR	258/316 (81.6)	489/546 (89.6)	10.17	0.001	1.72 (1.14-2.60)	0.010
BMI-defined overweight/obesity	193/316 (61.1)	394/546 (72.2)	10.82	0.001	1.55 (1.15-2.09)	0.004

Restricted to the 862 female participants. Values are n/N (%). Adjusted odds ratios compare women aged ≥50 years with women aged <50 years, adjusted for marital status and study site. Age ≥50 years is used only as a proxy for the menopausal transition; menopausal status was not ascertained.

Table S11. Prevalence of each adiposity definition by participant characteristic, with unadjusted omnibus tests

Characteristic	n	BMI-Defined Overweight/Obesity	Central Obesity (WC)	Central Obesity (WHR)	Central Obesity (WHtR)
Age group					
18-39 years	270	102 (37.8)	124 (45.9)	137 (50.7)	160 (59.3)
40-59 years	575	365 (63.5)	422 (73.4)	383 (66.6)	488 (84.9)
60-85 years	511	358 (70.1)	369 (72.2)	365 (71.4)	438 (85.7)
p-value		<0.001	<0.001	<0.001	<0.001
Sex					
Male	494	238 (48.2)	184 (37.2)	270 (54.7)	339 (68.6)
Female	862	587 (68.1)	731 (84.8)	615 (71.3)	747 (86.7)
p-value		<0.001	<0.001	<0.001	<0.001
Marital status					
Never-married	100	39 (39.0)	48 (48.0)	54 (54.0)	56 (56.0)
Ever-married	1256	786 (62.6)	867 (69.0)	831 (66.2)	1030 (82.0)
p-value		<0.001	<0.001	0.019	<0.001
Education level					
Pre-primary	123	75 (61.0)	96 (78.0)	92 (74.8)	101 (82.1)
Primary	561	343 (61.1)	385 (68.6)	377 (67.2)	452 (80.6)
Secondary	528	314 (59.5)	338 (64.0)	325 (61.6)	413 (78.2)
Tertiary	144	93 (64.6)	96 (66.7)	91 (63.2)	120 (83.3)

p-value		0.733	0.023	0.025	0.474
Monthly income					
≤150,000 MWK	931	566 (60.8)	625 (67.1)	617 (66.3)	740 (79.5)
>150,000 MWK	425	259 (60.9)	290 (68.2)	268 (63.1)	346 (81.4)
p-value		1.000	0.734	0.275	0.453
Diabetes duration					
<2 years	236	137 (58.1)	158 (66.9)	158 (66.9)	182 (77.1)
≥2 years	1120	688 (61.4)	757 (67.6)	727 (64.9)	904 (80.7)
p-value		0.372	0.909	0.601	0.243
Family history of diabetes					
No / do not know	768	455 (59.2)	500 (65.1)	491 (63.9)	613 (79.8)
Yes	588	370 (62.9)	415 (70.6)	394 (67.0)	473 (80.4)
p-value		0.187	0.038	0.262	0.828
Current treatment					
Lifestyle modification	32	20 (62.5)	23 (71.9)	17 (53.1)	26 (81.2)
Medication	1324	805 (60.8)	892 (67.4)	868 (65.6)	1060 (80.1)
p-value		0.991	0.729	0.203	1.000
Meal frequency					
21 meals/week	866	550 (63.5)	585 (67.6)	567 (65.5)	692 (79.9)
20 meals/week	46	35 (76.1)	35 (76.1)	27 (58.7)	39 (84.8)
<20 meals/week	444	240 (54.1)	295 (66.4)	291 (65.5)	355 (80.0)
p-value		<0.001	0.412	0.635	0.720
Late-evening eating behavior					
No	656	396 (60.4)	436 (66.5)	417 (63.6)	509 (77.6)
Yes	700	429 (61.3)	479 (68.4)	468 (66.9)	577 (82.4)
p-value		0.771	0.475	0.225	0.031
Physical activity					
Meets WHO recommendation	1166	682 (58.5)	774 (66.4)	757 (64.9)	923 (79.2)
Does not meet	190	143 (75.3)	141 (74.2)	128 (67.4)	163 (85.8)
p-value		<0.001	0.040	0.566	0.043

Sedentary behavior					
<8 hours/day	1240	755 (60.9)	837 (67.5)	812 (65.5)	996 (80.3)
≥8 hours/day	116	70 (60.3)	78 (67.2)	73 (62.9)	90 (77.6)
p-value		0.988	1.000	0.653	0.559
Depression					
Normal	988	609 (61.6)	676 (68.4)	634 (64.2)	793 (80.3)
Depressive symptoms	368	216 (58.7)	239 (64.9)	251 (68.2)	293 (79.6)
p-value		0.355	0.250	0.185	0.851
Anxiety					
Normal	866	528 (61.0)	585 (67.6)	543 (62.7)	691 (79.8)
Anxiety symptoms	490	297 (60.6)	330 (67.3)	342 (69.8)	395 (80.6)
p-value		0.943	0.986	0.010	0.770
Stress					
Normal	1193	723 (60.6)	811 (68.0)	775 (65.0)	958 (80.3)
Stress symptoms	163	102 (62.6)	104 (63.8)	110 (67.5)	128 (78.5)
p-value		0.690	0.328	0.585	0.669
Study site					
Mzuzu Central Hospital	202	135 (66.8)	142 (70.3)	131 (64.9)	167 (82.7)
Kamuzu Central Hospital	599	326 (54.4)	367 (61.3)	363 (60.6)	429 (71.6)
Zomba Central Hospital	555	364 (65.6)	406 (73.2)	391 (70.5)	490 (88.3)
p-value		<0.001	<0.001	0.002	<0.001

Values are n (%) of participants in that category who meet the adiposity definition; percentages are row-wise prevalences within the characteristic and are not expected to sum to 100% down a column. p-values are Pearson χ^2 tests across the categories of the characteristic. These comparisons are unadjusted and exploratory.

Table S12. Completeness of every variable used in the analysis

Variable	n observed	Missing, n (%)
BMI-defined overweight/obesity	1356	0 (0.0)
Central obesity (WC)	1356	0 (0.0)

Variable	n observed	Missing, n (%)
Central obesity (WHR)	1356	0 (0.0)
Central obesity (WHtR)	1356	0 (0.0)
BMI-WHtR phenotype	1356	0 (0.0)
Body mass index (kg/m^2)	1356	0 (0.0)
Waist circumference (cm)	1356	0 (0.0)
Hip circumference (cm)	1356	0 (0.0)
Waist-to-hip ratio	1356	0 (0.0)
Waist-to-height ratio	1356	0 (0.0)
Height (m)	1356	0 (0.0)
Weight (kg)	1356	0 (0.0)
Age (years)	1356	0 (0.0)
Weekly meal frequency (count)	1356	0 (0.0)
Age group	1356	0 (0.0)
Sex	1356	0 (0.0)
Marital status	1356	0 (0.0)
Education level	1356	0 (0.0)
Monthly income	1356	0 (0.0)
Diabetes duration	1356	0 (0.0)
Family history of diabetes	1356	0 (0.0)
Current treatment	1356	0 (0.0)
Meal frequency (categorical)	1356	0 (0.0)
Late-evening eating behavior	1356	0 (0.0)
Physical activity	1356	0 (0.0)
Sedentary behavior	1356	0 (0.0)

Variable	n observed	Missing, n (%)
Depression	1356	0 (0.0)
Anxiety	1356	0 (0.0)
Stress	1356	0 (0.0)
Study site	1356	0 (0.0)

There were no missing values on any analytic variable, so every model reported in the manuscript and in this supplement was fitted on the full analytic sample of 1356 participants and no case was dropped.

Table S13. Model diagnostics and goodness-of-fit for the four binary logistic models

**Outcome**	**n**	**Events, n (%)**	**Covariates**	**Max GVIF^(1/2df)**	**Nagelkerke R^2**	**Hosmer–Lemeshow χ^2 (df)**	**p-value**
BMI-defined overweight/obesity (BMI ≥25 kg/m²)	1356	825 (60.8%)	6	1.07	0.143	5.87 (df=8)	0.662
Central obesity, WC (≥94 cm men / ≥80 cm women)	1356	915 (67.5%)	6	1.07	0.346	7.71 (df=8)	0.462
Central obesity, WHR (≥0.90 men / ≥0.85 women)	1356	885 (65.3%)	7	1.33	0.075	6.68 (df=8)	0.572
Central obesity, WHtR (≥0.50)	1356	1086 (80.1%)	5	1.07	0.160	18.70 (df=8)	0.017

Generalized variance inflation factors, by model

Outcome	GVIF^(1/2df) for each covariate in that model

BMI-defined overweight/obesity (BMI ≥25 kg/m²)	Age group 1.05; sex 1.02; marital status 1.07; family history of diabetes 1.01; meal frequency 1.00; physical activity 1.01
Central obesity, WC (≥94 cm men / ≥80 cm women)	Age group 1.05; sex 1.03; marital status 1.07; education level 1.01; family history of diabetes 1.01; physical activity 1.01
Central obesity, WHR (≥0.90 men / ≥0.85 women)	Age group 1.05; sex 1.03; marital status 1.08; education level 1.01; current treatment 1.01; depression 1.33; anxiety 1.33
Central obesity, WHtR (≥0.50)	Age group 1.04; sex 1.01; marital status 1.07; late-evening eating behavior 1.00; physical activity 1.01<br>Note: the models for BMI-defined overweight/obesity and for WC- and WHR-defined central obesity showed no evidence of lack of fit. The model for WHtR-defined central obesity did (Hosmer–Lemeshow χ^2 = 18.70, df = 8, p = 0.017). With 80.1% of participants meeting the WHtR criterion and n = 1,356, the Hosmer–Lemeshow test is sensitive to small departures from fit; the result is nevertheless reported in Section 3.5 of the manuscript and the covariate associations for that outcome are interpreted with corresponding caution.

All four models converged on the full sample with no cases dropped. The number of covariates differs between outcomes because the adjustment set is screened separately for each (Table S3). Generalized variance inflation factors are reported because several covariates have more than two levels; GVIF^(1/2df) is on the scale of the square root of a conventional VIF and the largest value across all four models was 1.33, so there is no material multicollinearity. The Hosmer–Lemeshow test gave no evidence of lack of fit for the BMI-defined, WC-defined or WHR-defined outcomes. For the WHtR-defined outcome the test was significant (χ^2=18.70, df=8, p=0.017), indicating imperfect calibration; this model retains the fewest covariates, and the result is noted as a limitation rather than corrected by adding covariates post hoc.

Table S14. STROBE Statement - checklist of items that should be included in reports of cross-sectional studies

Section / Topic	Item	Recommendation	Reported in
Title and abstract	1(a)	Indicate the study's design with a commonly used term in the title or the abstract	Title; Abstract (Methods)
	1(b)	Provide in the abstract an informative and balanced summary of what was done and what was found	Abstract

Section / Topic	Item	Recommendation	Reported in
Introduction - Background/rationale	2	Explain the scientific background and rationale for the investigation being reported	Section 1, paragraphs 1-3
Introduction - Objectives	3	State specific objectives, including any prespecified hypotheses	Section 1, final paragraph
Methods - Study design	4	Present key elements of study design early in the paper	Section 2.1
Methods - Setting	5	Describe the setting, locations, and relevant dates, including periods of recruitment and data collection	Section 2.1
Methods - Participants	6	Give the eligibility criteria, and the sources and methods of selection of participants	Section 2.2
Methods - Variables	7	Clearly define all outcomes, exposures, predictors, potential confounders, and effect modifiers; give diagnostic criteria if applicable	Sections 2.4.1, 2.4.2, 2.5
Methods - Data sources/measurement	8	For each variable of interest, give sources of data and details of methods of assessment; describe comparability of assessment methods if there is more than one group	Sections 2.4, 2.5
Methods - Bias	9	Describe any efforts to address potential sources of bias	Sections 2.2, 2.4; Section 4.1
Methods - Study size	10	Explain how the study size was arrived at	Section 2.3
Methods - Quantitative variables	11	Explain how quantitative variables were handled in the analyses; if applicable, describe which groupings were chosen and why	Sections 2.4.1, 2.4.2, 2.5, 2.6; Table S9
Methods - Statistical methods	12(a)	Describe all statistical methods, including those used to control for confounding	Section 2.6; Table S3
	12(b)	Describe any methods used to examine subgroups and interactions	Section 2.6; Tables S6, S9, S10
	12(c)	Explain how missing data were addressed	Section 2.6; Table S12

Section / Topic	Item	Recommendation	Reported in
	12(d)	If applicable, describe analytical methods taking account of sampling strategy	Section 2.6 (study site as a parallel fixed-effect analysis); Table S2
	12(e)	Describe any sensitivity analyses	Section 2.6; Section 3.5; Tables S2, S6, S8, S9, S10
Results - Participants	13(a)	Report numbers of individuals at each stage of the study	Section 3.1
	13(b)	Give reasons for non-participation at each stage	Section 3.1 (33 refusals reported; reasons for the 100 post-consent exclusions are not disaggregated)
	13(c)	Consider use of a flow diagram	Numbers reported in text (Section 3.1); no flow diagram used
Results - Descriptive data	14(a)	Give characteristics of study participants and information on exposures and potential confounders	Section 3.1; Table 1; Table S11
	14(b)	Indicate number of participants with missing data for each variable of interest	Section 2.6; Table S12 (no missing data)
Results - Outcome data	15	Report numbers of outcome events or summary measures	Sections 3.1, 3.2; Table 1; Table S1
Results - Main results	16(a)	Give unadjusted estimates and, if applicable, confounder-adjusted estimates and their precision; make clear which confounders were adjusted for and why they were included	Sections 3.3, 3.4; Tables 2, 3; Table S3
	16(b)	Report category boundaries when continuous variables were categorized	Sections 2.4.1, 2.4.2, 2.5
	16(c)	If relevant, consider translating estimates of relative risk into absolute risk for a meaningful time period	Adjusted prevalence ratios and adjusted predicted prevalences, Tables S4 and S5
Results - Other analyses	17	Report other analyses done	Section 3.5; Tables S2, S6, S8, S9, S10

Section / Topic	Item	Recommendation	Reported in
Discussion - Key results	18	Summarize key results with reference to study objectives	Section 4, paragraph 1
Discussion - Limitations	19	Discuss limitations of the study, taking into account sources of potential bias or imprecision; discuss both direction and magnitude of any potential bias	Section 4.1
Discussion - Interpretation	20	Give a cautious overall interpretation of results considering objectives, limitations, multiplicity of analyses, results from similar studies, and other relevant evidence	Section 4; Section 5
Discussion - Generalizability	21	Discuss the generalizability (external validity) of the study results	Section 4.1
Other information - Funding	22	Give the source of funding and the role of the funders for the present study and, if applicable, for the original study on which the present article is based	Funding statement
